# Wheat F-box Protein TaFBA1 Positively Regulates Plant Drought Tolerance but Negatively Regulates Stomatal Closure

**DOI:** 10.3389/fpls.2019.01242

**Published:** 2019-10-10

**Authors:** Jie An, Qinxue Li, Junjiao Yang, Guangqiang Zhang, Zhongxian Zhao, Yunzhen Wu, Yong Wang, Wei Wang

**Affiliations:** State Key Laboratory of Crop Biology, Shandong Key Laboratory of Crop Biology, College of Life Sciences, Shandong Agricultural University, Tai’an, China

**Keywords:** ABA signaling pathway, protein interaction ABA receptor, wheat F-box protein, drought stress, stoma movement, antioxidant competence

## Abstract

The phytohormone abscisic acid (ABA) regulates plant growth and development, as well as responses to various stresses, such as salt and drought. The wheat *TaFBA1* gene, which encodes an F-box protein, was previously identified in our laboratory by homologous cloning. We previously found that *TaFBA1* expression was induced by ABA and drought stress. In this study, wild-type (WT), *TaFBA1* over-expressing (OEs), *TaFBA1* homologous gene mutants, and *TaFBA1* recovery (Rs) *Arabidopsis* plants were used. We found that the germination rate, the cotyledon greening rate, the root length, and the photosynthetic performance of TaFBA1 OE plants were better than those of WT under drought and ABA conditions, but mutant plants showed the opposite trend, and overexpression of TaFBA1 in mutants can recover their phenotype. In addition, TaFBA1 was found to be a negative regulator of ABA-induced stoma movement; mRNA transcription of certain ABA signaling-related genes was lower in TaFBA1 OE plants than in WT plants following ABA treatment. Further, we found that TaFBA1 can interact with RCAR1 (an ABA receptor) and ABI5. BiFC assay showed that TaFBA1 may interact with RCAR1 in the plasma membrane. In addition, accumulation of ROS and MDA in TaFBA1 OE plants was lower than that in the WT plants after ABA and drought treatments. Based on these results, we suggest that TaFBA1-regulated ABA insensitivity may be dependent on regulating ABA-mediated gene expression through interacting with RCAR1 and ABI5. Increased antioxidant competence and decreased ROS accumulation may be an important mechanism that underlies improved drought tolerance in TaFBA1 OE plants.

## Introduction

Drought stress is one of the most common environmental stresses in crops and affects the growth and development of plants. It may even lead to plant death in severe cases, thus reducing crop yield. Plants mainly exchange gas and water with the external environment through openings on leaf stomata, which modulates photosynthesis (Damour et al., 2010; Taylor et al., 2012). Under drought stress, to maintain their water balance, plants usually close their stomata to reduce transpiration. However, this also limits CO_2_ entry, which is detrimental to photosynthesis (Franks and Beerling, 2009). Stomatal closure reduces CO_2_ absorption under drought conditions, resulting in decreased dark reaction rate of photosynthesis, this is closely associated with cell damage caused by excessive accumulation of reactive oxygen species (ROS) in plants (Posch and Bennett, 2009; Juvany et al., 2014). By controlling the flow of water and CO_2_, stomata can allow plants to adapt to stress.

Abscisic acid (ABA) is an important plant hormone that regulates plant growth and development, such as embryo formation, seed dormancy and germination, seedling growth, lateral root development, and responses to various abiotic stresses including drought, high salt, and extreme temperature (Verslues and Zhu, 2005; Finkelstein, 2006; Fujii et al., 2007). Under drought conditions, the ABA concentration in plant roots increases, and the ABA signaling pathway is activated. This, in turn, activates various ion channels that regulate the movement of stoma and enzymes associated with physiological and biochemical reactions, eventually leading to stoma closure (Lee et al., 2009). To date, analysis of mutant lines has led to the characterization of many negative and positive regulators of ABA signaling in response to abiotic stresses (Hirayama and Shinozaki, 2007; Kim et al., 2011). Components of the ABA signaling pathways that have been identified include PYR/PYL/RCAR, clade A protein phosphatases type-2C (PP2Cs), and sucrose non-fermenting 1-related subfamily 2 protein kinases (SnRK2s), which phosphorylate transcription factors and ion channels to turn on ABA signaling (Ma et al., 2009; Park et al., 2009; Cutler et al., 2010). PP2Cs interacts with SnRK2s to inhibit their kinase activity and shut off the ABA signaling without ABA. When ABA is present, ABA receptor inhibits the activity of its integration with the PP2C, leading to SnRK2, which can be relieved from the inhibition state of PP2C. At the same time, activation of SnRK2 through phosphorylate modification ABA response of transcription factors, thereby switching ABA signaling (Hubbard et al., 2010).

Ubiquitin/26S proteasome system (UPS)-mediated protein degradation is an important mechanism for regulating the level and function of proteins in cells, including proteins in the ABA signaling pathway. UPS can degrade functional proteins with short half-lives, denatured and allosteric proteins, as well as structural proteins during emergency (Vierstra, 2009). The UPS consists of ubiquitin, E1 (ubiquitin-activated enzyme), E2 (ubiquitin-binding enzyme), E3 (ubiquitin-ligase), DUB (de-ubiquitylase), and 26S proteasome (Vierstra, 2003). The *Arabidopsis thaliana* genome contains more than 1,400 genes that encode components of the UPS, most of which (about 1,200) encode subunits of E3s (Lee and Kim, 2011). E3s in plants can be divided into the following types: the HECT domain family, the Ring/U-box domain family, the SCF (Skp1–Cullin–F-box) domain family, and the APC domain family (Cardozo and Pagano, 2004; Wiborg et al., 2008; Rotin and Kumar, 2009). The F-box protein is a key subunit protein in the SCF complex that is involved in responses to abiotic stresses (Zhang et al., 2009). It recognizes specific substrates and mediates protein–protein interactions during ubiquitination. Many studies suggested that its C-terminal structural domains can interact with proteins and can specifically identify substrates, such as WD40, leucine zipper (LRR), ARM, and zinc finger structures.

UPS plays an important role in the regulation of ABA signaling. For example, SCF type E3 ligase PHLOEM PROTEIN2-B11 inter-reacts with SnRK2.3 to promote its degradation (Cheng et al., 2017). The F-box protein RCAR3 INTERACTING F-BOX PROTEIN 1 (RIFP1) is an adaptor subunit of the SCF complex that can participate in RCAR3-mediated ABA signaling pathway to regulate ABA receptor RCAR3 degradation (Li et al., 2016a; Li et al., 2016b). DCAF-CUL4 E3 ubiquitin ligase assembled with ABA-hypersensitive DCAF1 (ABD1) is a negative regulator of ABA responses by directly binding to and affecting the degradation of ABI5 in the nucleus (Seo et al., 2014). The single subunit E3 ligase ABI3 INTERACTING PROTEIN 2 (AIP2) can adjust the stability of ABI3 protein; CUL4-based ligases and the RING E3 ligase KEEP ON GOING (KEG) can target ABI5 for degradation (Zhang et al., 2005; Lee et al., 2010; Liu and Stone, 2010).

A gene encoding the wheat F-box protein, *TaFBA1* (GenBank ID JN038382), was previously obtained in our laboratory by homologous cloning. We studied the functions of TaFBA1 in plant growth and stress tolerance by overexpressing it in tobacco. Study results showed that overexpression of TaFBA1 improves drought, salt, heat, and oxidative stress tolerance in transgenic tobacco plants (Zhou et al., 2014; Zhou et al., 2015; Kong et al., 2016; Zhao et al., 2017; Li et al., 2018). Results also showed that seed germination in transgenic tobacco that overexpresses TaFBA1 is less sensitive to ABA; stoma movement of TaFBA1-overexpressing tobacco was also insensitive to drought stress in our laboratory. Since ABA plays a major role in stomatal regulation, we wanted to determine: (1) whether TaFBA1 regulates ABA signaling pathway and/or ABA metabolism, (2) the physiological and molecular mechanism underlying TaFBA1-regulated ABA signal, (3) and how to improve the drought tolerance of transgenic plants with larger stomata opening under drought stress. In addition, the *Arabidopsis* mutant *atfbw2* and recombinant recovery plants were used. Further, we analyzed interactions between TaFBA1 and associated proteins in the ABA signaling pathway.

## Materials and Methods

### Gene Constructs and Generations of TaFBA1 Transgenic Plants

For the *TaFBA1* overexpression vector construct, the *TaFBA1* coding region was PCR-amplified using *TaFBA1*-F and *TaFBA1*-R primers (**Supplementary Table S1**). First, the *TaFBA1* coding region was cloned into the pENTR/D-TOPO vector (Invitrogen, China) for sequencing. It was then subcloned as an EcoRV fragment into the pEarleyGate101 vector, under the control of the CaMV 35S promoter. We performed the transformation of wheat using the particle bombardment method (Weeks et al., 1993; Wang et al., 2019), and wheat cv CB037 was used as background material. For the transformation of *Arabidopsis*, the construct was transferred into *Agrobacterium tumefaciens* 3101 and was then transformed into *Arabidopsis* Col-0 ecotype and mutant plants using *Agrobacterium*-mediated transformation. Homozygous transgenic plants selected in the T2 generation and confirmed in the T3 generation, *via* Kanamycin selection and PCR screening, PCR instrument T100 (Bio-Rad, United States) was used. Mature T3 seeds were used for subsequent experiments.

Homozygous T-DNA insertion mutants Salk_144548 (*atfbw2-4*), Salk_071588 (*atfbw2-5*), and Salk_765388 (*atfbw2-6*) were obtained from the *Arabidopsis* Stock Centre (http://arabidopsis.info/) and were verified by PCR (Zhao et al., 2017).

### Seed Germination and Plant Growth Assays

To assay the seed germination rate, approximately 25 surface-sterilized seeds from TaFBA1-overexpressing lines (OEs: OE4, OE5, OE6), mutants (*atfbw2-4*, *atfbw2-5*, *atfbw2-6*), recovery plants (Rs: R-4, R-5, R-6), and WT were germinated on half-strength Murashige–Skoog (1/2 MS) medium containing mannitol (150 mM, simulated drought condition), mannitol with Na_6_WO_4_ (0.1 mM, ABA synthetic inhibitor), or different concentrations of ABA (0, 1, 2 μM) under a 6-h light/8-h dark photoperiod at 22°C. Seed germination rates were evaluated by measuring root emergence after sowing for 1 week (Li et al., 2013a; Li et al., 2013b).

OEs, mutants, Rs, and WT seeds were germinated and allowed to grow on a 1/2 MS medium under normal conditions for 3 days. We were then transferred into 1/2 MS medium containing different concentrations of ABA, mannitol, or mannitol with Na_6_WO_4_ for 14 days. Plants were then imaged, and root length was measured.

### Photosynthetic Parameter Assays

Four-week-old plants grown in vermiculite were either treated with 2-μM ABA for 10 days, or were subjected to drought condition for 14 days. Ciras-3 portable photosynthetic apparatus was used in this study (PP SYSTEMS, United States). Net leaf photosynthetic rate (Pn), intercellular CO_2_ concentration (Ci), transpiration rate (E), and stomatal conductance (Gs) were measured according to methods outlined by Wang et al. (2015).

### Stomatal Aperture Measurements

Stomatal bioassays were performed as previously described (Tan et al., 2018). Leaves from 4-week-old *Arabidopsis* plants were treated with 2-μM ABA, and changes in stomatal aperture were examined within 2 h. The lower part of the leaf was then isolated and treated with opening solutions [50 mM CaCl_2_, 10 mM KCl, 10 mM MES–Tris (pH 6.15)]. Stomata was imaged under microscope AX10 (Zeiss, Germany) coupled to a camera; 30 stomatal apertures were examined for each treatment.

### Gene Expression Analysis by RT-PCR and Real-Time qPCR

Total RNA was then extracted from treated seedlings, and 2 mg of total RNA was for cDNA synthesis for RT-PCR (Bio-Rad, United States) in accordance with the manufacturer’s protocol (QuantiTect Reverse Transcription Kit; TIANGEN) (Kumar et al., 2017). RT-PCR analyses were conducted using gene-specific primers. To detect changes in gene expression after ABA treatment, a 25-μL reaction mixture was subjected to qRT-PCR using Super Real Premix Plus (TIANGEN, Beijing, China). Quantitative analysis was performed *via* a real-time PCR detection system using the CFX96 Touch™ (Bio-Rad, United States). *UBQ1* was used as the internal reference gene in all experiments. Relative expression levels of detected genes were calculated using the delta-delta Ct method. Information from all genes in the qRT-PCR experiments are listed in **Supplementary Table S1**.

### Protein Interaction Assay

Yeast two-hybrid (Y2H) assays were performed as previously described (Li et al., 2018). Complementary DNA (cDNA) fragments of RCAR1/ABI5 were cloned into pGBKT7 or pGADT7 vectors. Similarly, cDNA fragments of *TaFBA1* and *AtFBW2* were cloned into pGBKT7 or pGADT7 vectors. The lithium acetate-mediated transformation method was used to cotransform two proteins present in different vectors into the yeast strain Y2H Gold. Cotransformed Y2H was applied to SD-Leu-Trp, SD-Leu-Trp-His, and SD-adenine-His-Leu-Trp. Yeast growth was observed in a 30°C incubator for 3 to 5 days to verify the interactions between bait and prey proteins.

For *in vivo* bimolecular fluorescence complementation (BiFC) assays, *TaFBA1* was attached to the cloned vector and was fused to the C-terminal of YFP; the *RCAR1* fragment was attached to pENTR/D-TOPO and was fused to the YFP N-terminal. We transferred the fusion plasmid with YFP N-terminal and YFP C-terminal into *A. tumefaciens* (strain GV3101), which was then injected into *Nicotiana benthamiana* leaves. YFP fluorescence in infiltrated leaves was observed under a fluorescence microscope LSM880 (Zeiss, Germany) for 48 h.

In the LCI assay, *TaFBA1* was attached to the C-terminal of LUC and the *RCAR1* fragment was fused to the LUC N-terminal. We transferred the fusion plasmid with LUC N-terminal and LUC C-terminal into *A. tumefaciens* (strain GV3101), which was then injected into *N. benthamiana* leaves. The fluorescent signal was observed with a whole-body imaging system IVIS200 (Xenogen United States).

### H_2_O_2_ Detection, Superoxide Anion Radical (O^−^_2_) Measurements, Histochemical Staining of O^−^_2_, and Determination of Chlorophyll and MDA Contents

Histochemical O^−^_2_ staining, as well as determination of H_2_O_2_ and O^−^_2_ content were performed as previously described (Zhou et al., 2014).

Malondialdehyde (MDA) levels were measured according to protocols described by Li et al. (2013a, 2013b).

For the determination of chlorophyll and MDA contents in transgenic wheat, 2-week-old CB037 and transgenic wheat leaves were soaked in 10-µM methyl viologen (MV) solution for 48 h in a chamber at 25°C with a 16-h light/8-h dark cycle at a relative humidity of 75% to 80%, and then the materials were used to measure the chlorophyll and MDA contents. The detailed steps were adopted by Yang et al. (2009).

### Statistical Analysis

Statistical analysis was using one-way ANOVA implemented in the SPSS software 13. Significant difference was detected at *P* < 0.01. The data were presented as the mean ± standard error (SE) of three independent experiments.

## Results

### Involvement of TaFBA1 in Drought Stress Tolerance of *Arabidopsis* Plants

**Figure 1:**
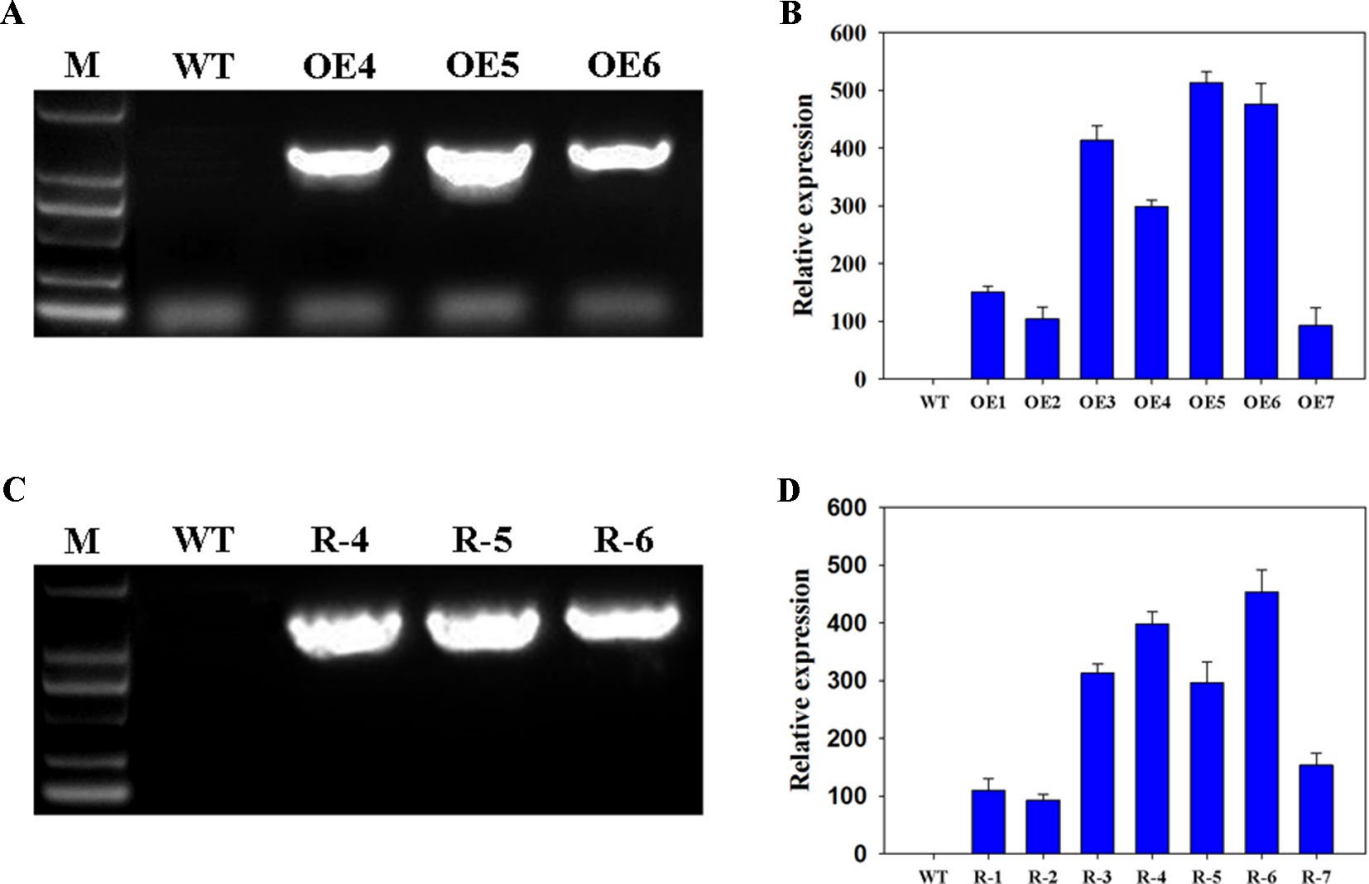
Confirmation of *TaFBA1* transgenic lines by genomic PCR and real-time PCR. Genomic PCR **(A**, **C)** and qRT-PCR **(B**, **D)** of TaFBA1 overexpression in *Arabidopsis thaliana* lines **(A**, **B)** and *TaFBA1* homozygous gene mutant recovery lines **(C**, **D)**.

To study the functions of wheat *TaFBA1* in drought tolerance and stoma movement, the transgenic *Arabidopsis* plants with overexpressed *TaFBA1* (OEs), OE4, OE5, and OE6 were used in this experiment (**Figures 1A, B** and **Figure S1**). Previously, we found *Arabidopsis* gene homologous to wheat *TaFBA1*, namely, *AtFBW2*, three mutants, *atfbw2-4*, *atfbw2-5*, and *atfbw2-6*, were obtained. It was found that *AtFBW2* and *TaFBA1* have similar gene expression patterns when responding to salt stress (Zhao et al., 2017). In this experiment, we transfected *TaFBA1* with *atfbw2* to obtain recovery plants (Rs), R-4, R-5, and R-6 lines were used in subsequent experiments (**Figures 1C, D**).

**Figure 2:**
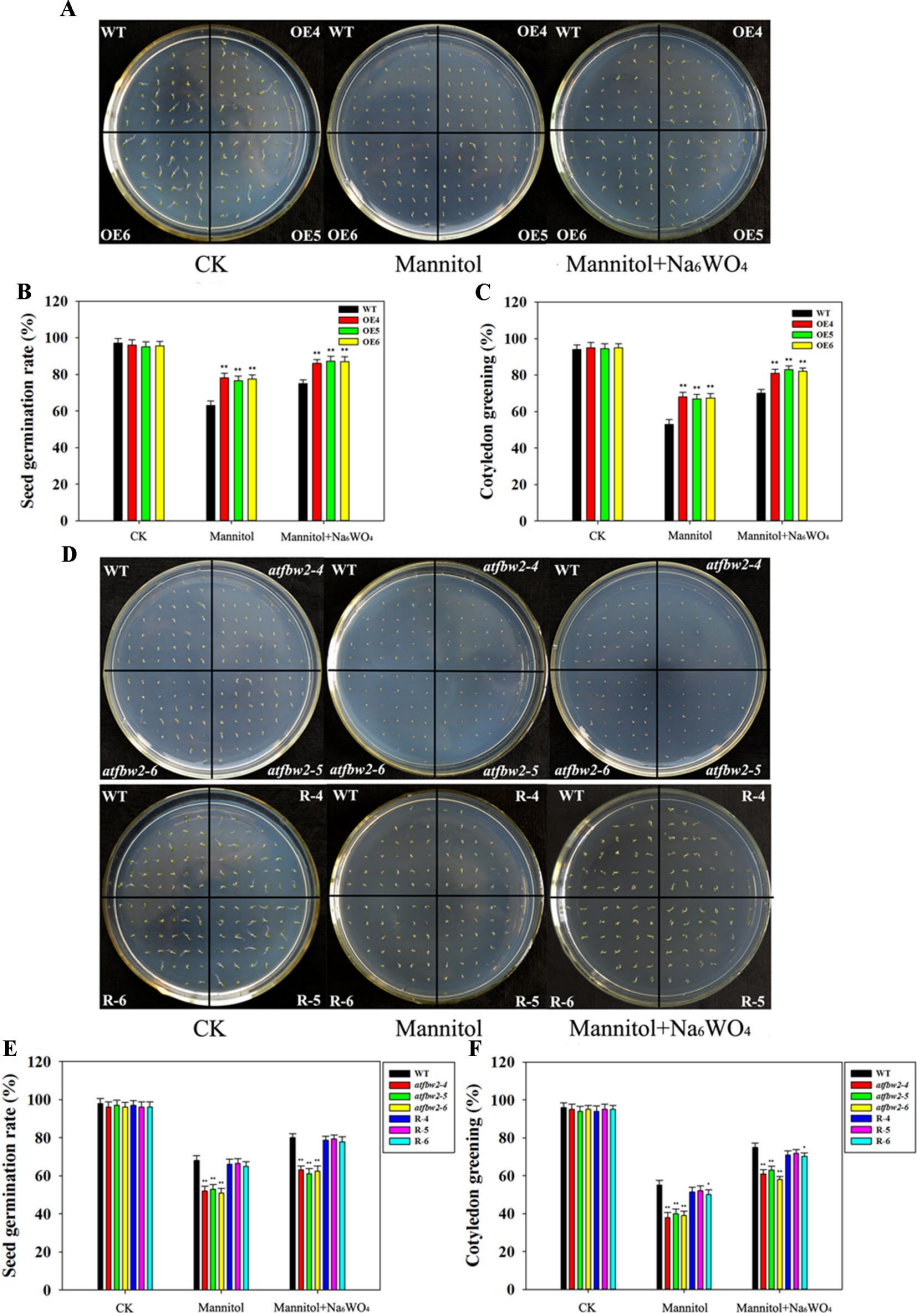
Germination and cotyledon greening rates of *Arabidopsis* plants under mannitol and mannitol + Na_6_WO_4_ conditions. **(A)** Germination phenotype, **(B)** germination rates, and **(C)** cotyledon greening rates of TaFBA1 overexpressing lines (OE4, OE5, OE6) and WT plants. **(D)** Germination phenotype, **(E)** germination rates, and **(F)** cotyledon greening rates of WT, mutant (*atfbw2-4*, *atfbw2-5*, *atfbw2-6*) and recovery lines (R-4, R-5, R-6). Germination rates were measured 4 days after sowing, cotyledon greening rates were measured at 7 days post-sowing. Data represent the mean ± SE of three biological replicates. *P < 0.05; **P < 0.01.

First, we tested the drought tolerance of *Arabidopsis* plants, including OEs (OE4, OE5, and OE6), mutants (*atfbw2-4*, *atfbw2-5* and *atfbw2-6*), and Rs (R-4, R-5, and R-6) plants. Mannitol was used to induce drought stress, and Na_6_WO_4_ (an ABA biosynthetic inhibitor) was used to inhibit ABA biosynthesis (Li et al., 2013a; Li et al., 2013b). As shown in **Figures 2A–C**, germination and cotyledon greening rates of OEs seeds were earlier than those of WT under mannitol conditions mutants that showed delayed rates, whereas Rs plants showed similar rates with WT (**Figures 2D–F**). We then added Na_6_WO_4_ into the culture medium containing mannitol and found that Na_6_WO_4_ could alleviate the inhibition of mannitol on seed germination and cotyledon greening rates in all *Arabidopsis* plants. However, no significant differences were observed in the extent of inhibition between OEs, WT, mutants, and Rs plants (**Figure 2**).

**Figure 3:**
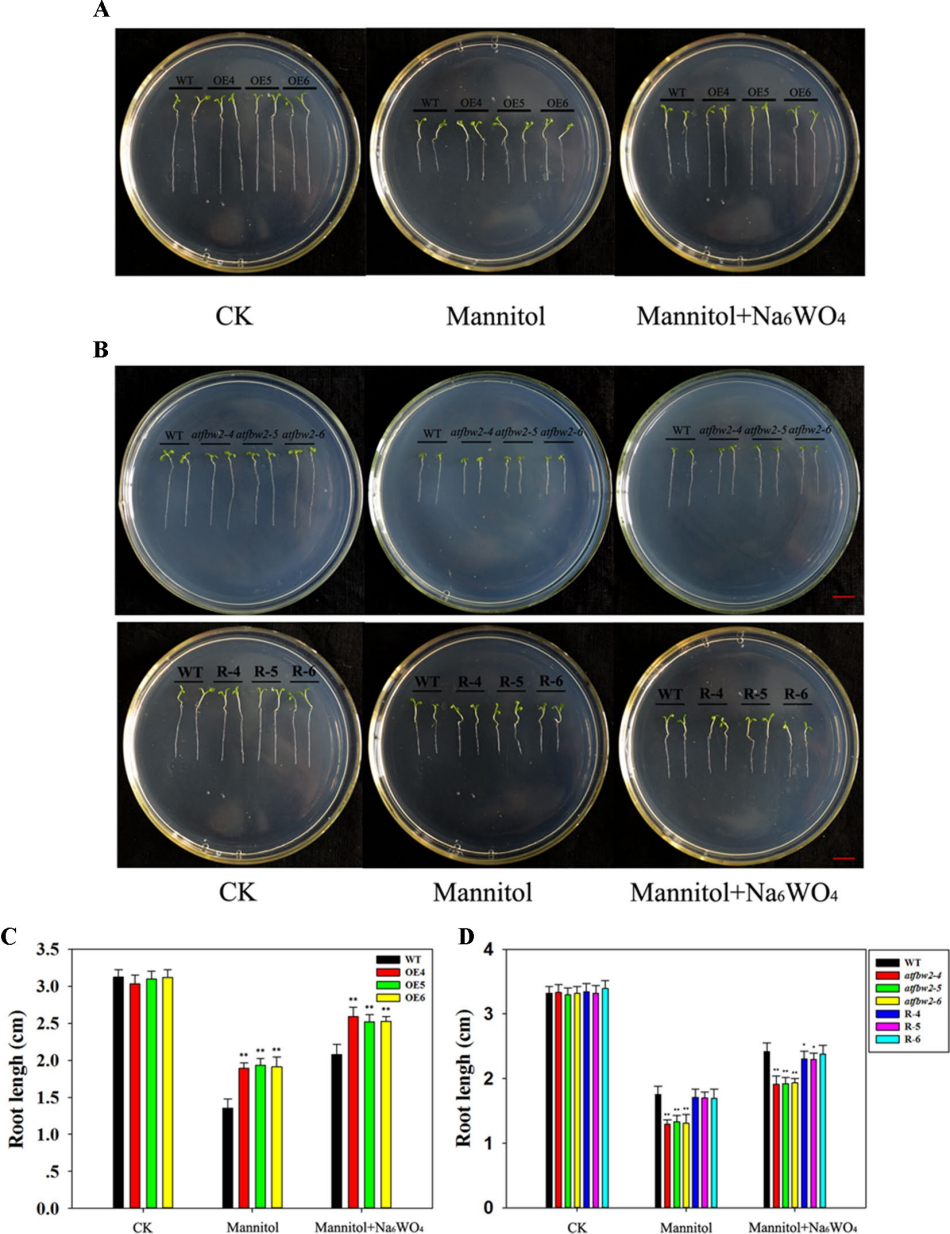
Root growth of *Arabidopsis* plants under mannitol and mannitol + Na_6_WO_4_ conditions. **(A)** Root growth phenotype and **(C)** root length statistics of WT and TaFBA1 overexpressing lines (OE4, OE5, OE6). **(B)** Root growth phenotype and **(D)** root length statistics of WT, mutant (*atfbw2-4*, *atfbw2-5*, *atfbw2-6*), and recovery plants (R-4, R-5, R-6). Root lengths were measured 5 days following transfer of 3-day-old seedlings from MS medium to plates with or without mannitol and mannitol + Na_6_WO_4_. Data represent the mean ± SE of three biological replicates. *P < 0.05; **P < 0.01.

Germinating seeds grown in 1/2 MS were transferred to medium containing mannitol and mannitol + Na_6_WO_4_. It was found that roots of WT plants were shorter than those of OE plants (**Figures 3A, C**) a week following mannitol treatment. Moreover, mutants exhibited a delay in root development with respect to the WT. Root length of Rs plants was longer than that of the mutants (**Figures 3B, D**). Similar to the results for germination and cotyledon greening (**Figure 2**), the addition of Na_6_WO_4_ alleviated the inhibition of mannitol on root growth. However, no significant differences were observed in the extent of inhibition between OEs, WT, mutants, and Rs plants (**Figure 3**).

We also measured ABA levels. Data showed that ABA levels in WT, OEs, and mutants plants were not significantly different following drought treatment, except for *atfbw2-4* (**Figure S2**).

**Figure 4:**
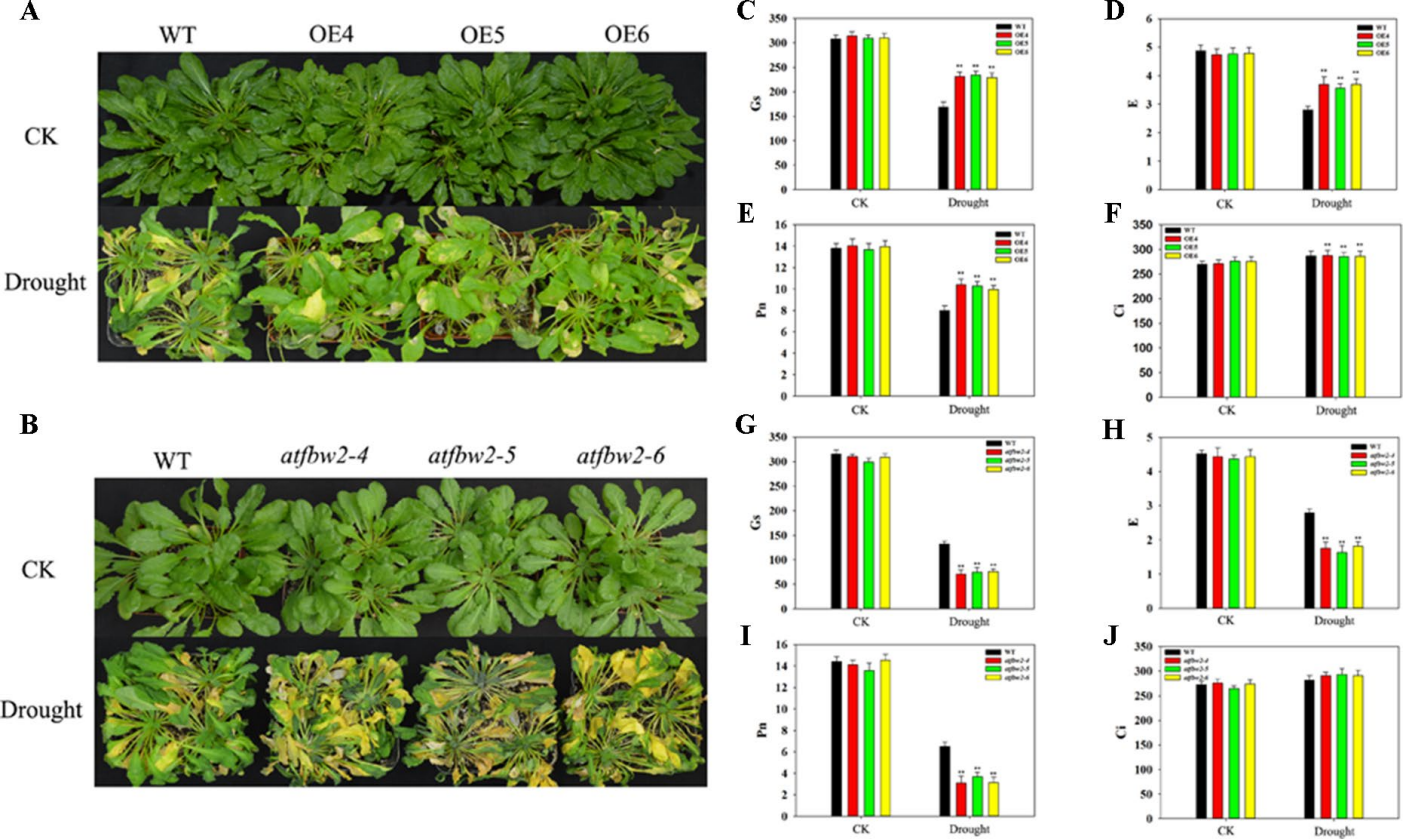
Growth and photosynthetic capacity of *Arabidopsis* plants under drought stress. **(A)** Phenotypes of WT and TaFBA1-overexpressing lines (OE4, OE5, OE6), and **(B)** WT and mutant (*atfbw2-4*, *atfbw2-5*, *atfbw2-6*) lines. **(C**, **G)** Stomatal conductance (Gs), **(D**, **H)** transpiration rate **(E)**, **(E**, **I)** net photosynthetic rate (Pn), and **(F**, **J)** intercellular CO_2_ concentration (Ci) of *Arabidopsis* leaves in *TaFBA1*-overexpression **(C**–**F)** and mutants lines **(G**–**J)** under normal and drought stress treatments. Data represent the mean ± SE of three biological replicates. *P < 0.05; **P < 0.01.

Further, 30-day-old OEs and WT *Arabidopsis* plants were grown in pots, and soil was allowed to dry in the absence of water for 11 days. There was no significant difference in growth between WT, OEs, mutants, and Rs plants under normal conditions. However, we observed severe leaf wilting in mutants lines under drought conditions; lighter leaf wilting was observed in OEs plants; Rs plants showed comparable characteristics to those of WT plants (**Figures 4A, B** and **Figure S3A**). We then rehydrated them and found that the OE plants had better rehydration ability, whereas the mutant plants had poor rehydration ability (**Figure S3B, C**).

We also examined photosynthetic parameters of all plants. There was no difference in the Pn of plants under normal conditions. Following drought treatment, the Pn of WT decreased more than that of OEs plants; mutant plants showed the greatest reduction in Pn. E and Gs responses were consistent with those of Pn. However, Ci demonstrated the opposite trend to Pn, being slightly higher in OE than WT plants (**Figures 4C–J**). These results indicated that overexpression of TaFBA1 could improve the photosynthetic performance of transgenic plants under drought stress.

The results above (**Figures 2**–**4** and **Figures S2**, **S3**) indicated that overexpression of TaFBA1 conferred higher tolerance to drought stresses in *Arabidopsis*. Drought tolerance of mutant plants was found to be weakened, which suggested that TaFBA1 plays a positive regulatory role in drought stress tolerance. ABA biosynthesis was not involved in the improved drought tolerance of TaFBA1-overexpressing plants.

### Effect of TaFBA1 on ABA Signaling-Mediated Growth and Stomatal Movement in *Arabidopsis*

**Figure 5:**
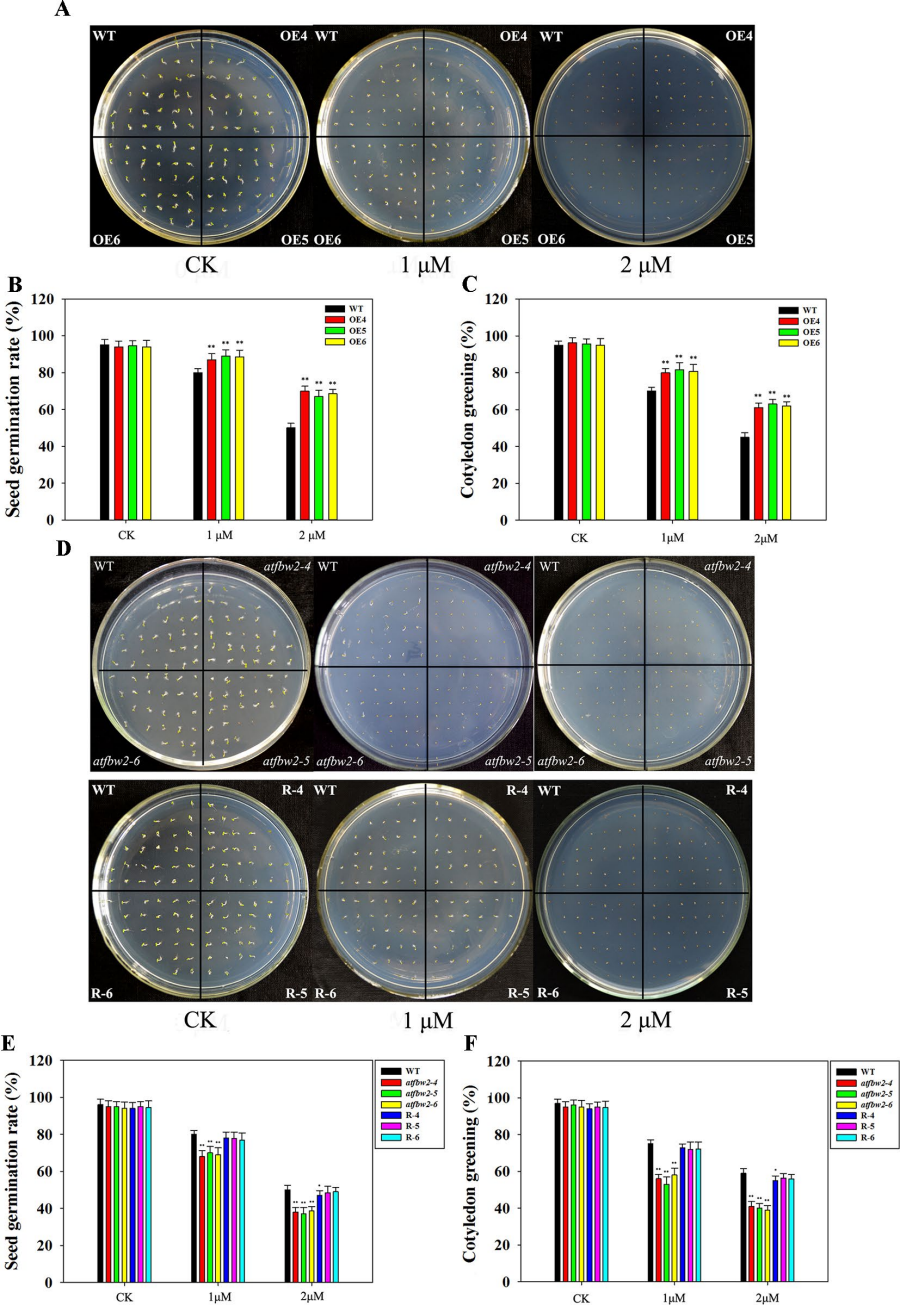
Germination and cotyledon greening rates of *Arabidopsis* plants following ABA treatment. **(A)** Germination phenotype, **(B)** germination rates, and **(C)** cotyledon greening rates of OEs (OE4, OE5, OE6) and WT lines in different concentrations of ABA. **(D)** Germination phenotype, **(E)** germination rates, and **(F)** cotyledon greening rates of WT, mutant (*atfbw2-4*, *atfbw2-5*, *atfbw2-6*), and recovery plants (R-4, R-5, R-6). Germination rates were measured 4 days after sowing, and cotyledon greening rates were measured at 7 days after sowing. Data represent mean ± SE of three biological replicates. *P < 0.05; **P < 0.01.

We have previously induced *TaFBA1* expression *via* ABA treatment. In this study, seeds from WT, OEs, mutant, and Rs plants were spread on 1/2MS medium with or without 1- or 2-μM ABA. There were no significant differences in the germination and cotyledon greening rates between OEs and WT *Arabidopsis* seeds under normal conditions without ABA. Following ABA treatment, seed germination and cotyledon greening of all plants were significantly inhibited; however, OEs were less inhibited than the WT, especially in the 2-μM ABA medium (**Figures 5A–C**). Mutants exhibited greater ABA-induced inhibition than did WT plants; the difference between them was more evident with increased ABA concentration. The germination and cotyledon greening rate of Rs plants were higher than those of mutants and were comparable to those of WT plants (**Figures 5D–F**).

**Figure 6:**
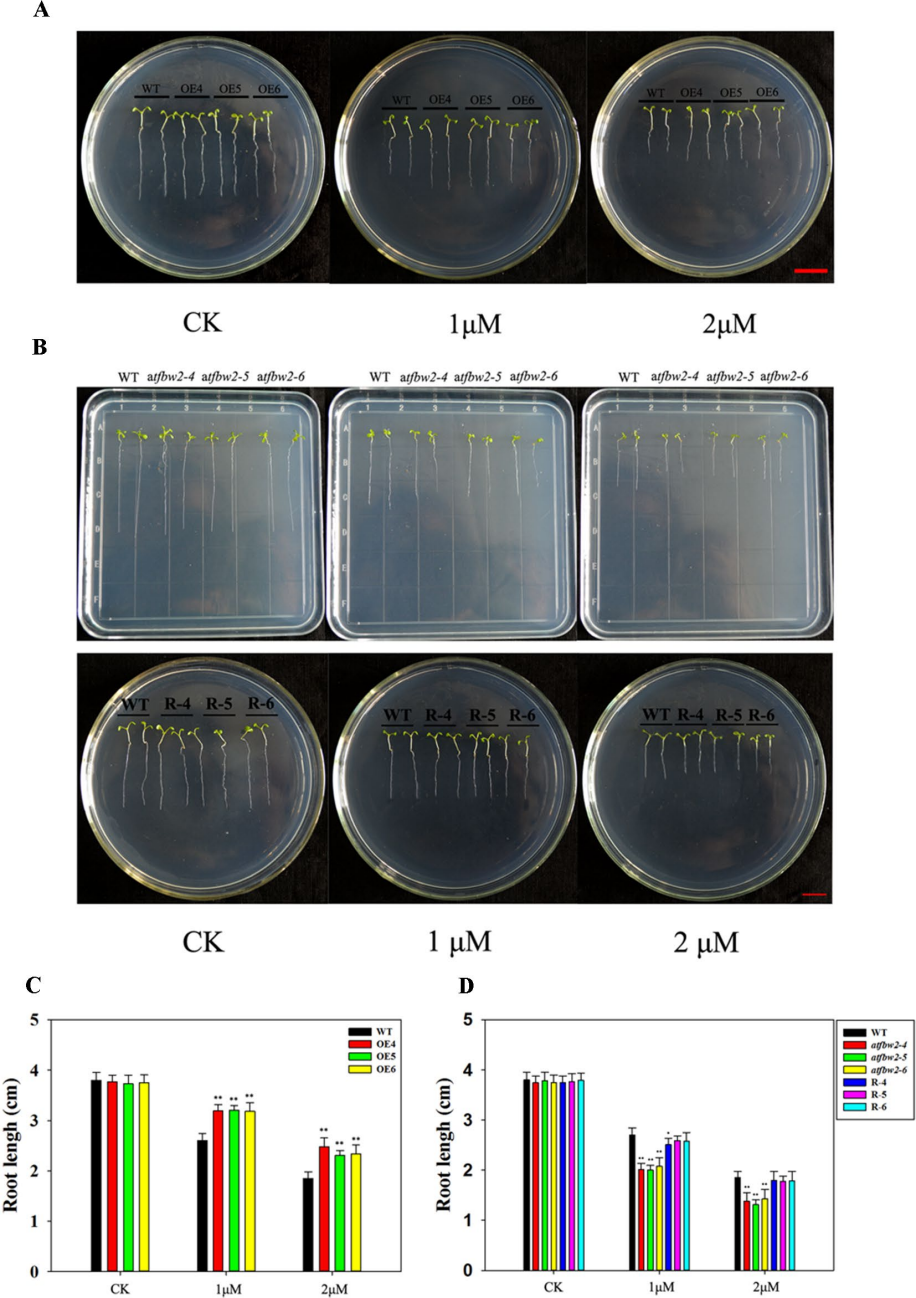
Root growth of *Arabidopsis* plants following ABA treatment. **(A)** Root growth phenotype and **(C)** root length statistics of WT and TaFBA1 overexpressing lines (OE4, OE5, OE6). **(B)** Root growth phenotype and **(D)** root length statistics of WT, mutant (*atfbw2-4*, *atfbw2-5*, *atfbw2-6*), and recovery plants (R-4, R-5, R-6). Root lengths were measured 5 days after transfer of 3-day-old seedlings from MS medium to plates with or without ABA. Data represent the mean ± SE of three biological replicates. *P < 0.05; **P < 0.01.

Similar to the germination and cotyledon greening rates, root growth of all plants was suppressed by ABA treatment. OEs plants showed much longer roots than those of WT plants. With increasing ABA concentrations, the differences between OEs and WT plants became more evident (**Figures 6A, C**). In addition, the roots of mutant plants were shorter than those of the WT in 1/2MS medium containing 1-μM ABA; this difference was even more pronounced with 2-μM ABA. The root length of R plants was similar to that of the WT plants (**Figures 6B, D**).

**Figure 7:**
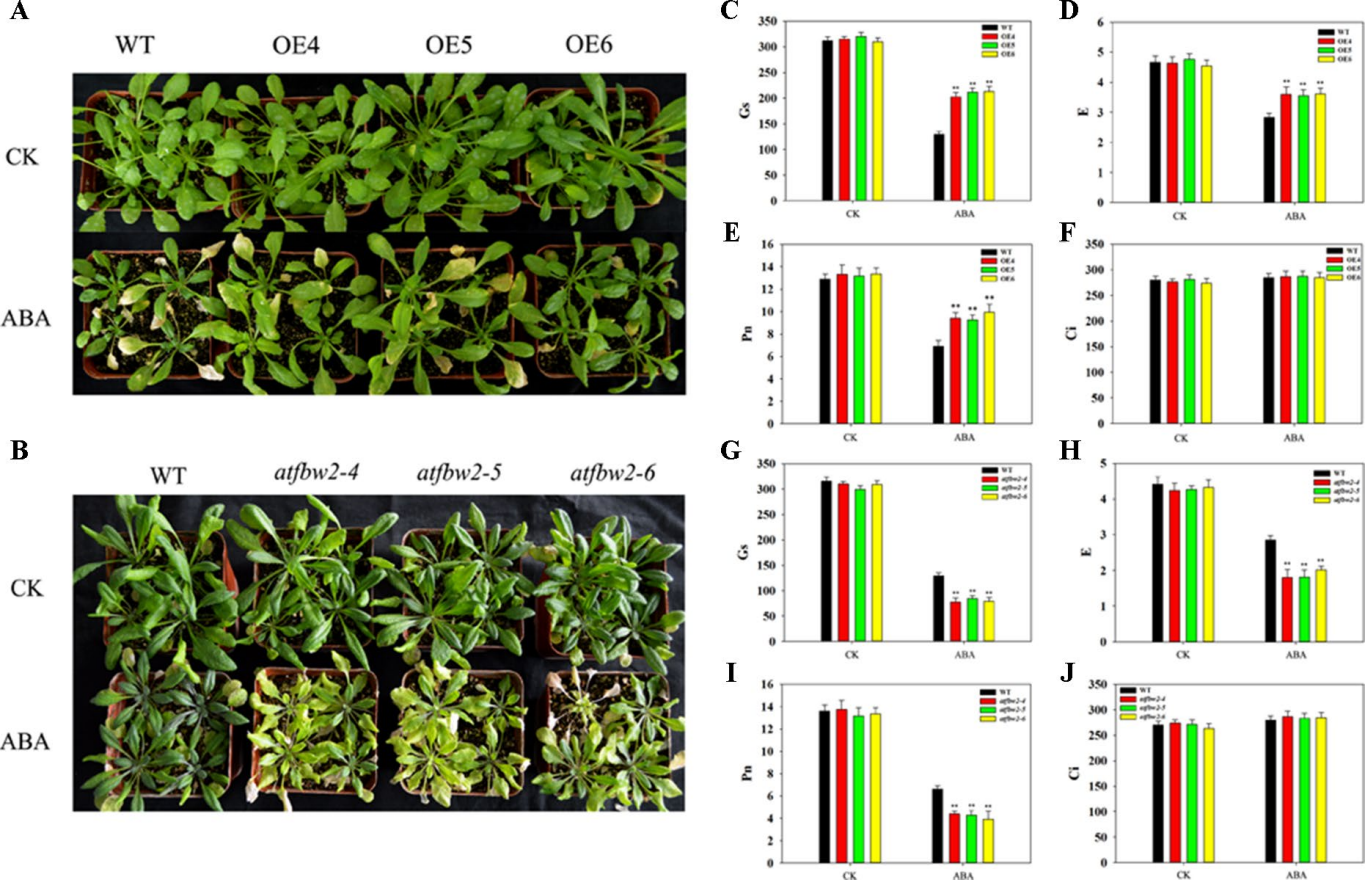
Growth and photosynthetic capacity of *Arabidopsis* plants after ABA treatment. **(A)** Phenotype of WT and *TaFBA1*-overexpressing plants (OE4, OE5, OE6), **(B)** WT and mutants (*atfbw2-4*, *atfbw2-5*, *atfbw2-6*) *Arabidopsis* lines 10 days after ABA treatment. **(C**–**J)** Photosynthetic parameters of *Arabidopsis* plant leaves after ABA treatment, including **(C**, **G)** stomatal conductance (Gs), **(D**, **H)** transpiration rate **(E)**, **(E**, **I)** net photosynthetic rate (Pn), and **(F**, **J)** intercellular CO_2_ concentration (Ci). Data represent mean ± SE of three biological replicates. *P < 0.05; **P < 0.01.

Further, 30-day-old *Arabidopsis* plants of OEs, mutants, Rs, and WT plants were exposed to ABA. There were no significant differences in the growth performance among them under normal conditions. However, we observed more severe leaf wilting in mutant lines than in OEs plants; the growth performance of Rs plants was similar to that of WT under ABA condition (**Figures 7A, B** and **Figure S4**).

We also examined photosynthetic parameters of OEs, mutants, and WT *Arabidopsis* plants under ABA conditions. There were no differences in the Pn of all plants under normal condition. Following ABA treatment, the Pn of OEs plants was gradually decreased, mutant plants showed the greatest reduction in Pn (**Figures 7E, I**). Other photosynthetic parameters, such as E and Gs, displayed similar behavior to that of Pn (**Figures 7C, D, G, H**). Ci showed the opposite trend to Pn, parallel to what was observed under drought stress (**Figures 7F, J**).

The above results indicated that TaFBA1 overexpression reduces ABA sensitivity of *Arabidopsis* plants, and that ABA sensitivity of the *TaFBA1* homologous gene mutant is increased compared with that of WT plants. However, ABA sensitivity of mutant plants can be compensated by TaFBA1 overexpression. TaFBA1 is, therefore, a negative regulator of the ABA signaling pathway.

**Figure 8:**
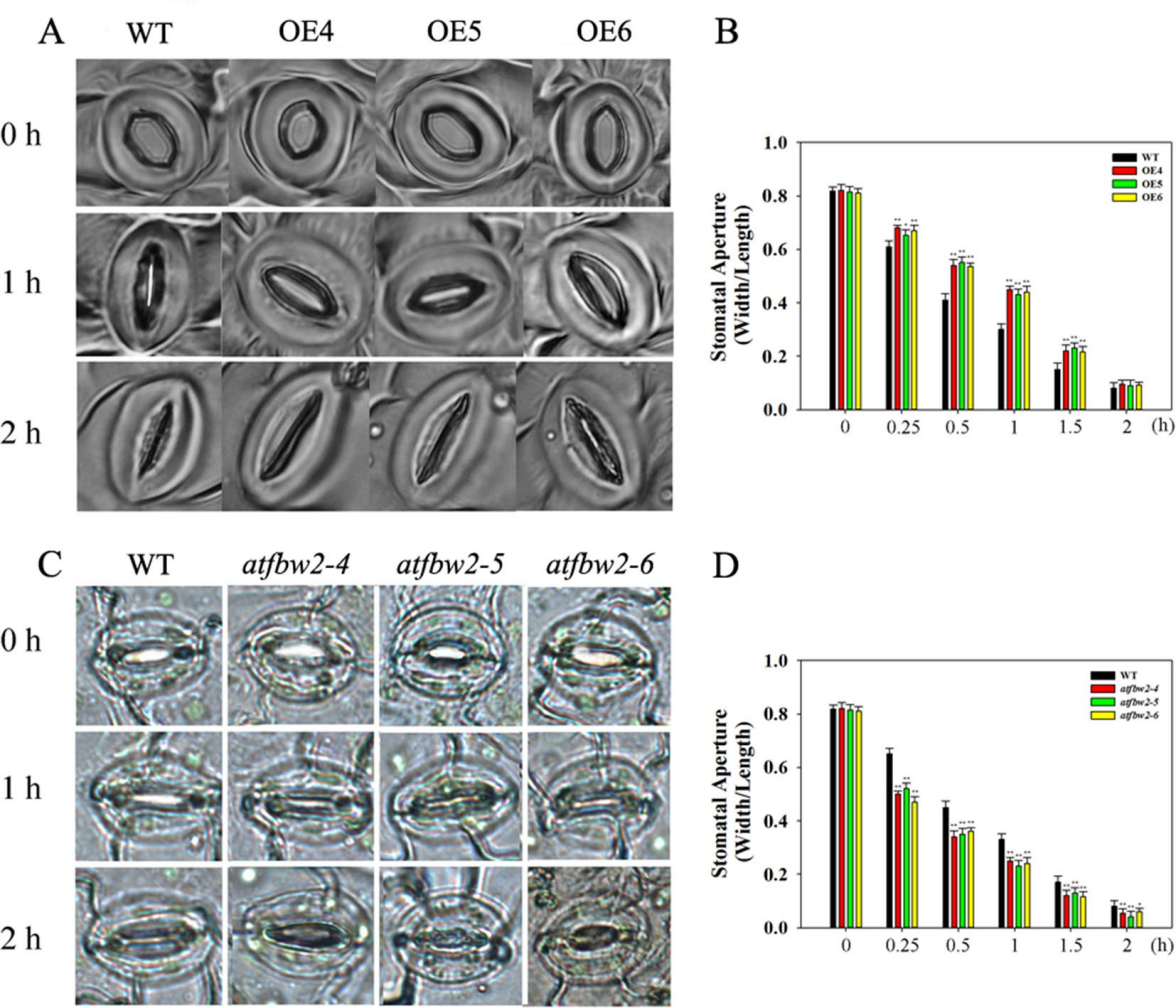
Stomatal closure response to ABA treatment in *Arabidopsis* plants. Stomatal closure phenotype of WT, OEs (OE4, OE5, OE6) **(A)**, and WT, mutants (*atfbw2-4*, *atfbw2-5*, *atfbw2-6*) **(C)**. Stomatal aperture of WT and overexpression of *TaFBA1* **(B)**, WT and mutants (*atfbw2-4*, *atfbw2-5*, *atfbw2-6*) **(D)** at different time points. Four-week-old *Arabidopsis* seedlings were treated with or without ABA for 2 h. Stomatal apertures (the ratio of width to length) were measured. Data represent the mean ± SE of three biological replicates. *P < 0.05; **P < 0.01.

We also examined stomatal movement of OEs, mutants, and WT plants within 2 h after ABA treatment. There were no significant differences in stomata aperture among OEs, mutants, and WT plants under normal condition. ABA treatment resulted in stomatal closure in all lines; however, stomatal aperture was dramatically smaller in mutants than in the WT. In addition, stomatal opening in OEs plants was significantly larger than that of WT plants (**Figures 8A, C**). We also examined stomatal opening responses 2 h following ABA treatment at different time points. As shown in **Figures 8B, D**, stomatal closure of OEs plants was slower than WT, but mutants faster than WT. After 2 h, stomata were closed in all plants.

These results demonstrated that TaFBA1 overexpression can inhibit ABA-induced stomatal closure in *Arabidopsis* plants, which further suggested that TaFBA1 plays a negative regulatory role in the ABA signaling pathway.

### Effects of TaFBA1 on Expression of ABA-Related Genes to ABA Treatment

**Figure 9:**
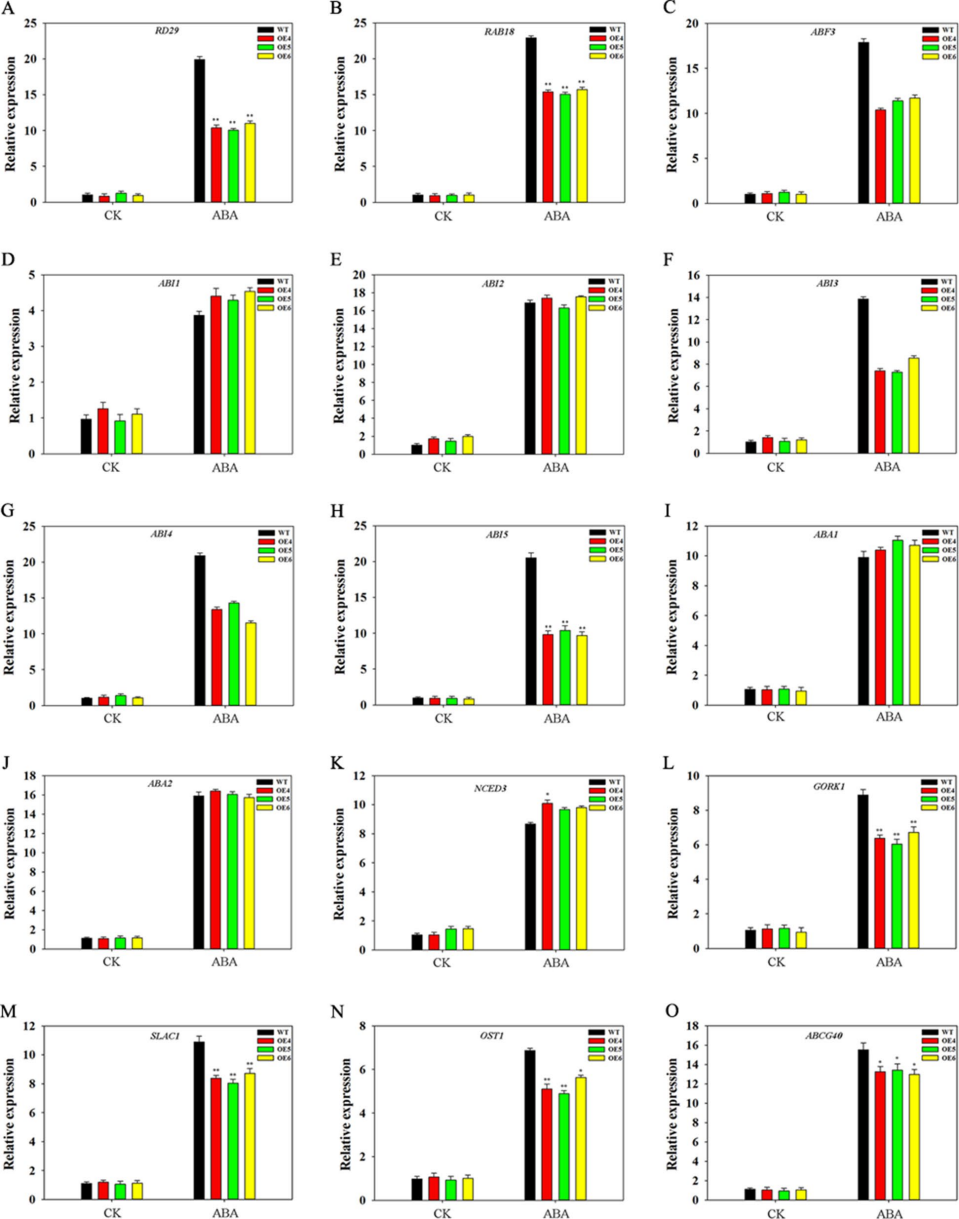
Expression patterns of ABA-associated genes in WT and TaFBA1-overexpressing (OE4, OE5, OE6) *Arabidopsis* plants under ABA treatment. **(A**–**H)** Expression levels of ABA-response genes, including *RD29*, *RAB18*, *ABF3*, *ABI1*, *ABI2*, *ABI3*, *ABI4*, *ABI5*. **(I**–**K)** Expression levels of ABA-synthesize associated related genes, including *ABA1*, *ABA2* and *NCED3*. **(L**–**O)** Expression of genes involved in stomatal movement, including *SLAC1*, *OST1*, *GORK1*, and *ABCG40*. Data represent mean ± SE of three biological replicates. *P < 0.05; **P < 0.01.

We analyzed the effect of TaFBA1 on expression of ABA-related genes. OEs and WT seedlings (10-day-old) germinated on 1/2 MS medium with or without 2-μM ABA were used. As shown in **Figure 9**, all genes were upregulated by ABA treatment in both WT and OEs plants. However, different gene expression levels between WT and OEs were observed under ABA treatment. Expression levels of some ABA response genes, including *RD29, RAB18, ABF3, ABI3, ABI4,* and *ABI5*, were lower in OEs plants than in WT under ABA treatments. However, no significant differences were observed in the expression levels of *ABI1* and *ABI2* in WT and OEs plants. We also examined expressions of ABA synthesis-associated genes (*ABA1*, *ABA2*, *NCED3*); results indicated that ABA upregulated transcription levels of these genes, but expression levels were comparable between WT and TaFBA1 OEs plants under ABA treatments (**Figures 9I–K**). We further measured transcriptional profiles of several genes associated with stomatal movement. Therein, *GORK1* and *SLAC1* (**Figures 9L, M**) encode key cation and anion channels that modulate the turgor of guard cells (Xu et al., 2016); *OST1* (**Figure 9N**) encodes the component that links upstream signals to ionic channels (Xu et al., 2016); *ABCG40* (**Figure 9O**) transports ABA from the apoplasm into the guard cell and facilitates ABA-mediated stomatal movement (Qiao et al., 2016). The expression of all these genes was found to be upregulated by ABA; gene expression levels were lower in the OEs lines than in the WT plants during ABA treatment.

### Interaction Between TaFBA1 and ABA Signaling Proteins

**Figure 10:**
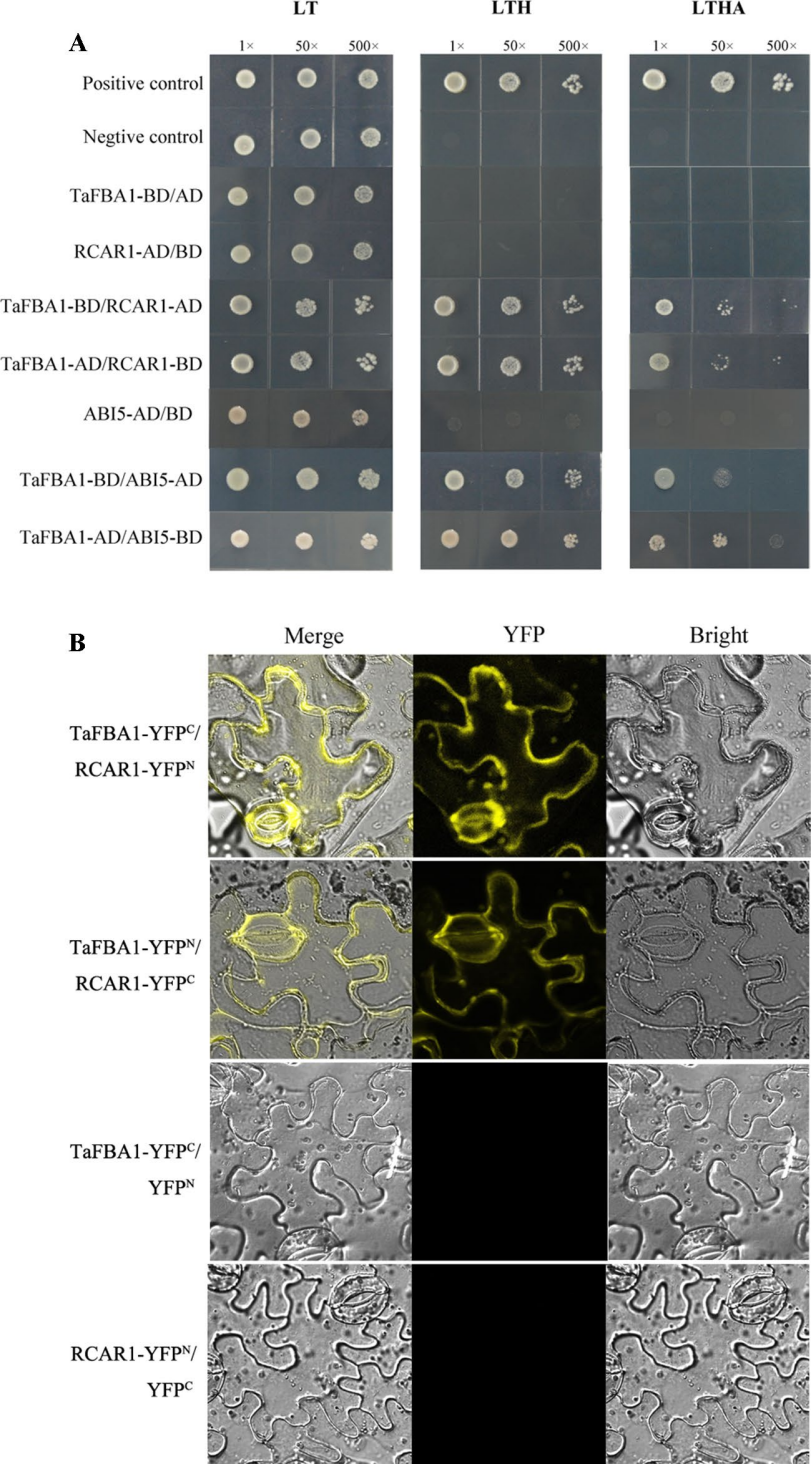
The interactions of TaFBA1 with RCAR1 (an ABA receptor) and ABI5. **(A)** Interactions of TaFBA1 with RCAR1 and ABI5 were analyzed *via* the yeast two-hybrid assay. Interaction between pGADT7-324 and pGBKT7-332, pGADT7-322 and pGBKT7-332, were used as a positive and negative controls, respectively. LT, LYH, and LTHA represent the selective media SD-Leu-Trp, SD-Leu-Trp-His, and SD-Leu-Trp-His-Ade, respectively. **(B)** BiFC assays were used to determine the interaction between TaFBA1 and RCAR1 using yellow fluorescence signal. nYFP denotes expression of the YFP N-terminal fusion construct. cYFP denotes expression of the YFP C-terminal fusion construct.

To identify how TaFBA1 takes part in the regulation of the ABA signaling pathway, we performed Y2H screening with TaFBA1 as the bait. As indicated by **Figure 10A**, TaFBA1-BD/RCAR1-AD, TaFBA1-AD/RCAR1-BD, TaFBA1-BD/ABI5-AD, and TaFBA1-AD/ABI5-BD can survive on SD-Leu-Trp-His and SD/-His/-Ade/-Trp/-Leu medium. This suggested that TaFBA1 can interact with RCAR1 (an ABA receptor) and ABI5 (ABA response protein). On the other hand, TaFBA1 did not interact with ABI2 and ABF3 (two ABA response protein, data not shown).

We further verified the interaction between TaFBA1 and RCAR1 through BiFC assays *in vivo*. The C-terminus of YFP was fused to TaFBA1 (YFP^C^-TaFBA1), and the N-terminus of YFP was fused to RCAR1 (YFP^N^-RCAR1); the construct was co-transfected into *N. benthamiana* leaf cells through *Agrobacterium* infiltration. A strong YFP signal was detected in the plasma membrane of leaf cells, whereas the negative control failed to yield any fluorescent signal (**Figure 10B**). This suggested that TaFBA1 may interact with RCAR1 on the plasma membrane. In the LCI assay, significant LUC activity was detected in leaves co-expressed with TaFBA1-LUC^N^ and LUC^C^-RCAR1 proteins, whereas no LUC activity was observed with the co-expression of TaFBA1-LUC^N^ and LUC^C^ (**Figure S5**).

In addition, Y2H was used to verify whether the *Arabidopsis* TaFBA1 homologous protein AtFBW2 could also interact with RCAR1 and ABI5, the results showed that AtFBW2 RCAR1 but not with ABI5 (**Figure S6**).

### Involvement of TaFBA1 in ROS Accumulation and Antioxidant Competence Under ABA and Drought Treatments

**Figure 11:**
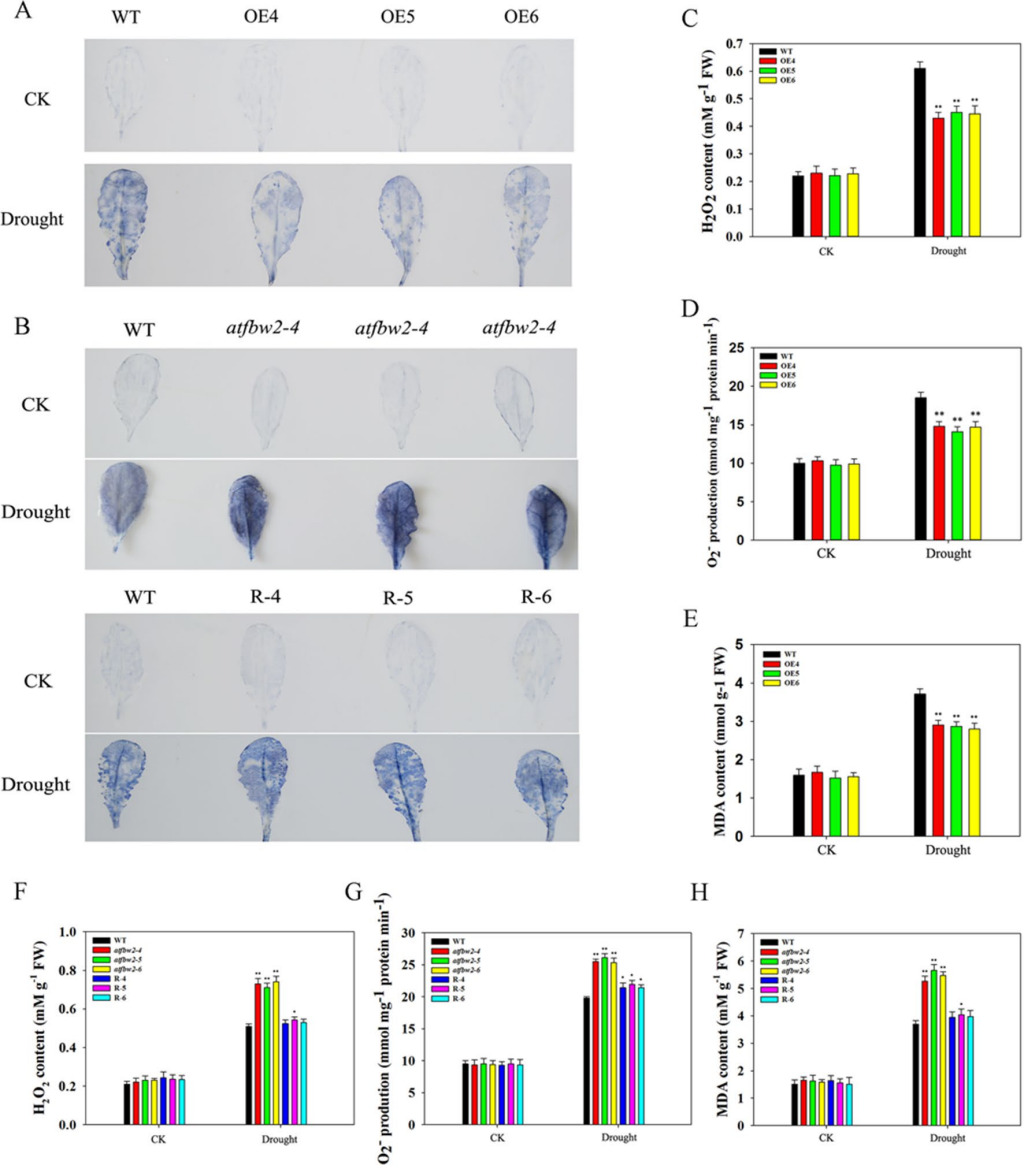
ROS accumulations and MDA content in *Arabidopsis* plants under drought stress. O_2_^−^ accumulations were detected by histochemical staining using NBT in leaves of **(A)** WT and OEs (OE4, OE5, OE6), as well as **(B)** WT, mutant (*atfbw2-4*, *atfbw2-5*, *atfbw2-6*) and recovery (R-4, R-5, R-6) *Arabidopsis* plants undergoing drought stress. Quantitative analysis of O_2_^−^ **(D**, **G)** and H_2_O_2_ **(C**, **F)**. MDA contents in WT and TaFBA1-overexpressing lines (OE4, OE5, OE6) **(E)**, as well as **(H)** WT, mutant (*atfbw2-4*, *atfbw2-5*, *atfbw2-6*), recovery plants (R-4, R-5, R-6) under drought stress. Data represent the mean ± SE of three biological replicates. *P < 0.05; **P < 0.01.

**Figure 12:**
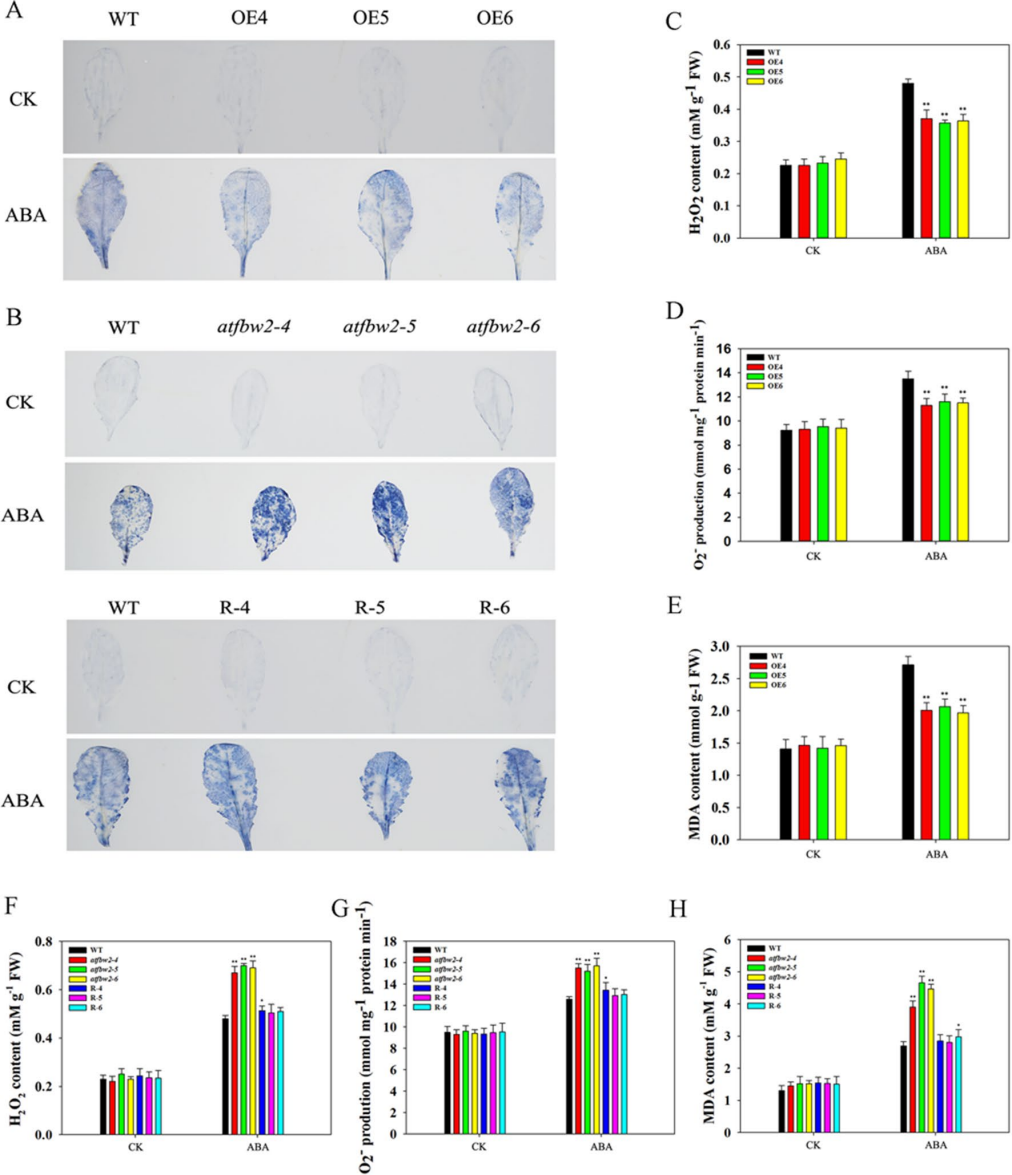
ROS accumulations and MDA content in *Arabidopsis* plants following ABA treatment. O_2_^−^ accumulations were detected by histochemical staining using NBT in the leaves of **(A)** WT and OEs (OE4, OE5, OE6) plants, as well as in **(B)** WT, mutant (*atfbw2-4*, *atfbw2-5*, *atfbw2-6*) and recovery (R-4, R-5, R-6) *Arabidopsis* plants. Quantitative analysis of O_2_^-^ **(D**, **G)** and H_2_O_2_ **(C**, **F)** were also conducted. MDA contents in **(E)** WT and TaFBA1-overexpression lines (OE4, OE5, OE6). **(H)** MDA contents in WT, mutant (*atfbw2-4*, *atfbw2-5*, *atfbw2-6*) and recovery plants (R-4, R-5, R-6). Data represent mean ± SE of three biological replicates. *P < 0.05; **P < 0.01.

It is known that adverse stress induces ROS accumulation (Dinakar et al., 2012). We measured levels of H_2_O_2_ and O^–^_2_ in WT, OEs, mutant, and Rs plants under drought and ABA conditions. First, we visualized O^–^_2_ accumulation *via* NBT staining, which was indicated by a blue color. Under normal conditions, O^–^_2_ levels were comparable among WT, OEs, mutant, and Rs plants. Following ABA and drought treatments, O^–^_2_ was produced in large quantities. As compared with WT plants, the blue color in OE plants was lighter. On the other hand, mutant plants exhibited darker color; light blue color was observed in Rs plants (**Figures 11A, B** and **12A, B**). We also conducted quantitative analysis on H_2_O_2_ and O^–^_2_ content under normal conditions and after ABA and drought stress treatments. Similar to NBT staining results, no significant differences in H_2_O_2_ and O^–^_2_ levels were detected between all plants under normal conditions. When subjected to ABA and drought stress, mutant plants accumulated greater H_2_O_2_ and O^–^_2_ than WT plants. However, TaFBA1-supplemented R plants exhibited greater reduction in H_2_O_2_ and O^–^_2_ accumulation than that in mutant plants, whereas OEs plants displayed the least H_2_O_2_ and O^–^_2_ accumulation (**Figures 11C, D, F, G** and **12C, D, F, G**).

MDA is one of the most important products of membrane lipid peroxidation, and its production can aggravate membrane damage. As shown in **Figures 11E, H** and **12E, H**, MDA content in OEs plants was lower than that of WT after ABA and drought treatments; mutant plants showed the opposite trend, TaFBA1-supplemented R plants exhibited lower MDA content than that of mutants. From these results, we suggest that TaFBA1 may improve the antioxidant capacity of *Arabidopsis* plants under drought and ABA stress and reduce damage to cell membranes.

Further, the antioxidant phenotype of transgenic wheat showed that, after 10-μmol MV treatment, the leaves of *TaFBA1* overexpression lines (OE2, OE6) were whitened more slightly than that of CB037, whereas they had no significant difference under normal growth condition. Contrarily, the leaves of the *TaFBA1*-RNAi lines (R4, R5) had more severe damage than CB037, but that difference was not very obvious (**Figures S7A**). Besides, under the normal growth condition, the five lines had similar total chlorophyll contents. However, when exposed to 10-μmol MV, the total chlorophyll contents of all the lines were decreased, and the OE lines had a higher chlorophyll contents than that in CB037. The RNAi lines had a slight decrease of chlorophyll contents compared with CB037 (**Figures S7B**). We also examined the MDA contents of five lines in wheat. After MV treatment, all the lines had increased MDA contents. The differences among CB037, OEs, Rs were similar to the total chlorophyll contents (**Figures S7C**). The data above suggested that overexpression *TaFBA1* could increase the antioxidant ability in wheat.

## Discussion

### TaFBA1 Positively Regulates Plant Drought Tolerance Independent of ABA Biosynthesis

Drought stress is an important environmental stress factor that affects agricultural production. Droughts cause plant dehydration, which leads to changes in various physiological processes, such as destruction of cell membrane structure, weakened photosynthesis, blocked metabolism, which ultimately impact plant growth and development (Bartels and Sunkar, 2005). When plants are exposed to adverse conditions, such as drought stress, endogenous ABA levels increase, which subsequently induces stress-related gene transcription (Zhu, 2002). Our previous studies showed that the wheat F-box gene *TaFBA1* is involved in plant stress tolerance. It was shown that TaFBA1 overexpression improves drought, salt, and high-temperature stress tolerance of transgenic tobacco plants (Kong et al., 2016; Zhao et al., 2017; Li et al., 2018). We also observed that ABA could induce expression of the *TaFBA1* gene in wheat (Zhou et al., 2014). However, the Gs of transgenic plants was greater than that of the WT under stress conditions; this usually leads to more water loss but greater CO_2_ absorption.

To understand the involvement of ABA in TaFBA1-regulated stomatal movement and plant stress tolerance, WT, TaFBA1 overexpressing (OEs), *TaFBA1* homologous gene mutants, and TaFBA1 recovery (Rs) *Arabidopsis* plants were used (**Figure 1**, and **Figure S1**). As shown in the results in **Figures 2** and **3**, the germination rate, cotyledon greening, and root length of TaFBA1 OEs plants were all greater than those of WT plants under drought conditions; those of *atfbw2* mutants were reduced under drought conditions. We also found that growth and Pn of grown OEs plants were greater than those of WT plants (**Figure 4**). This indicated that TaFBA1 positively regulates plant stress tolerance. Following the addition of Na_6_WO_4_ under drought conditions, the germination rate and root length of WT and transgenic *Arabidopsis* seeds all showed various degrees of recovery. This suggested that inhibition of ABA levels could alleviate plant growth inhibition under drought conditions; however, differences in these parameters were maintained among OEs, mutants, Rs, and WT plants even with Na_6_WO_4_ addition (**Figures 2**, **3**). We further examined ABA levels in OEs, mutants, and WT plants, and no significant differences were observed between these plants, with the exception of *atfbw2-4* (**Figure S2**). The expression of ABA synthesis-associated genes (*ABA1*, *ABA2*, *NCED3*) was comparable between OEs and WT plants under ABA treatment. These results suggested that TaFBA1 may not be involved in ABA biosynthesis. It was reported earlier that the F-box protein MAX2 can respond to abiotic stress conditions in *Arabidopsis*; *max2* mutant was found to be hypersensitive to drought stress (Bu et al., 2014), which is consistent with our results. Therefore, TaFBA1 may positively regulate plant drought tolerance, independent of ABA biosynthesis.

### TaFBA1 Negatively Regulates Sensitivity of *Arabidopsis* Plants to ABA

ABA is involved in plant growth and stress tolerance, which includes inhibiting seed germination, cotyledon greening, and seedling growth, but inducing stomatal closure under drought stress (Zhu, 2002). In *Arabidopsis*, a negative regulator of ABA signaling pathway is drought resistance (DOR), which is an F-box protein; it forms specific interactions with *Arabidopsis* SKP1-LIKE 14 (ASK14) and CUL1. DOR mutation promotes stomatal closure, which in turn increases plant tolerance to drought (Zhang et al., 2008). ABA-responsive FBA domain-containing protein 1 (AFBA1), an F-box protein involved in ABA signaling, regulates plant drought resistance in *Arabidopsis*. In mutant *afba1*, stomatal closure is blocked, water loss rate is increased, and the plant is sensitive to drought (Kim et al., 2017). We showed that when *Arabidopsis* plants were treated with ABA, they exhibit similar phenotypes to those treated under drought conditions. Following ABA treatment, the seed germination rate, cotyledon greening, and root length of OEs plants were improved with respect to those of WT plants; mutant plants exhibited poorer phenotypes as compared with those of WT (**Figures 5**, **6**). When grown *Arabidopsis* plants were treated ABA, both the phenotype and photosynthetic performance of OEs seedlings were enhanced; these characteristics were worsened in mutant plants as compared with those of WT plants (**Figure 7** and **Figure S4**). Taken together, these results suggested that TaFBA1 negative regulates sensitivity of *Arabidopsis* plants to ABA treatments.

As shown in **Figure 4B**, Gs was bigger in OE plants as compared with that of the WT. This suggested possible involvement of ABA signaling in TaFBA1-regulated drought tolerance. Stomatal movement is regulated not only by various environmental conditions, such as light, carbon dioxide (CO_2_) (Hubbard et al., 2012), nitric oxide (NO) (Sugiyama et al., 2012), and temperature, but also by a variety of plant hormones, such as ABA, ethylene, methyl jasmonate, and brassinosteroids (BRs) (Suhita et al., 2004; Haubrick et al., 2006; An et al., 2008). ABA is the main plant hormone that induces stomatal closure and is recognized as an important response factor under drought stress. Although early and high fast stomatal closure affects photosynthesis and, hence, biomass production, late and slow stomatal closure can rapidly exhaust available soil water, which results in yield loss through reduced post-anthesis water use (Saradadevi et al., 2017). In our study, stomal changes in WT and transgenic plants were also analyzed within 2 h of ABA treatment. As shown in **Figure 8**, overexpression of TaFBA1 decreased sensitivity of OEs plants to ABA. In addition, stoma closure of homologous gene mutants was more sensitive to ABA than WT, suggesting that TaFBA1 is a negative regulator in ABA-induced stoma movement.

### Regulating Gene Expression by Interaction With RCAR1 and ABI5 May Be Involved in TaFBA1-Regulated ABA Insensitivity in Transgenic *Arabidopsis* Plants

ABA regulates the expression of genes, such as those encoding transcription factors and other signaling molecules (Cutler et al., 2010) to modulate plant growth phenotype and abiotic stress tolerance. There are nine ABA-related b-zip transcription factors in *A. thaliana*, and *abi5* mutants specifically show a certain degree of ABA insensitivity during seed germination (Jakoby et al., 2002). ABA can induce the expression of stoma-related genes. Further, stomatal closure requires regulation of ion balance through a series of ion channels or transporters in cells, which is mostly regulated by ABA signaling. In this study, ABA treatment upregulated expression of some genes associated with the ABA signaling pathway, such as *ABF3, ABI3, ABI4,* and *ABI5*, ABA downstream response genes *RAB18* and *RD29*, greater increase in gene expression levels was observed in WT plants than in OEs plants. Transcription of certain genes that mediate stomatal movement was also upregulated by ABA treatment, including *GORK1*, *SLAC1, OST1*, and *ABCG40* (Qiao et al., 2016; Xu et al., 2016), but increased levels were less in OEs plants than WT (**Figures 9L–O**). These results implied that TaFBA1 may regulate stomatal movement by regulating the expression of genes related to relevant components in the ABA signaling pathway.

We next wanted to determine how TaFBA1 regulates gene expression. It was reported that ABRE binds to the transcription factor ABF3, a nuclear gene that is a key regulator of the ABA signaling pathway (Fujita et al., 2005). Some E3 ligases were involved in the ubiquitination and degradation of ABA receptors (RCARs). Targeted degradation of ABA receptors RCAR3/PYL8 is mediated by the ubiquitin ligase substrate adaptor DDB1 ASSOCIATED1 (DDA1) in *Arabidopsis* (Irigoyen et al., 2014). The single subunit RING-type E3 ubiquitin ligase RING FINGER OF SEED LONGEVITY1 (RSL1) targets PYL4 and PYR1 ABA receptors in the plasma membrane to modulate ABA signaling (Bueso et al., 2014). Similarly, the DWD protein AtRAE1 (RNA export factor1 in *Arabidopsis*), which may act as a substrate receptor of CUL4-DDB1 E3 ligase, is an interacting partner of RCAR1/PYL9 (Li et al., 2017). Our previous study indicated that TaFBA1 may be a member of the SCF complex and interacts with other proteins in SCF, such as SKP1 and Cullin. It is also involved in the formation of the SCF complex. TaFBA1 also interacts with the wheat stress response protein TaASRP1 (Li et al., 2018). Y2H and LCI assays indicated the interaction between TaFBA1 and the ABA receptor RCAR1 (**Figure S5**). Furthermore, BiFC showed that that their interaction was located on the plasma membrane (**Figure 10**). We also observed that TaFBA1 interacts with the ABA response protein ABI5 using Y2H technology. AtFBW2 could also interact with RCAR1 but not with ABI5 (**Figure S6**).

These results suggested that TaFBA1 may regulate expression of ABA signaling genes by interacting with RCAR1 and ABI5, which may be involved in TaFBA1-regulated ABA insensitivity of transgenic *Arabidopsis* plants.

### Increasing Antioxidant Competence and Decreasing ROS Accumulation May Be Important Mechanisms That Underlies Improved Drought Tolerance in TafBa1 Overexpressing Transgenic Plants

Drought induces ROS production in plants. Excessive ROS accumulation leads to metabolic imbalance and oxidative damages. These damages include detrimental effects on the structure and function of cells, such as inhibition of chloroplast development, reduced photosynthesis rate, rapid increase in MDA, destruction of membrane integrity, and even cell death (Mittler, 2002; Apel and Hirt, 2004). The transgenic strategy could, therefore, improve the plant’s antioxidant competence. For instance, when the soybean miR172c is overexpressed, H_2_O_2_ and O^–^_2_ accumulations were significantly reduced following either ABA or water deficit treatments (Li et al., 2016a; Li et al., 2016b). Many secondary messengers participate in the regulation of ABA signaling as well, such as ROS. Chen and Gallie (2004) studied the ABA signal transduction network of guard cell movement in rapeseed and found that ROS plays a leading role in the regulation of guard cell movement. The transcription factor HAT1-impaired stomatal closure may be caused by changed H_2_O_2_ levels in guard cells (Tan et al., 2018). In our previous studies, TaFBA1 overexpression improved antioxidant capacity of transgenic tobacco under drought, high salt, heat, and MV stresses (Zhou et al., 2014; Zhou et al., 2015; Kong et al., 2016; Zhao et al., 2017; Li et al., 2018). In this study, we found that after ABA and drought treatment, H_2_O_2_ accumulation in OE lines was lower than that in the WT; mutants showed greater H_2_O_2_ accumulation, and R plants exhibited lower accumulation than that in mutants (**Figures 11A, B** and **12A, B**). Quantitative analysis of H_2_O_2_ and O^–^_2_ (**Figures 11C, D, F, G** and **12C, D, F, G**) suggested that ABA and drought dramatically induce H_2_O_2_ and O^–^_2_ accumulation in WT plants, overexpression of TaFBA1 significantly decreased H_2_O_2_ and O^–^_2_ accumulations; mutants displayed the opposite trend. Decreased ROS accumulations in OEs plants resulted in less membrane lipid peroxidation, as indicated by reduced MDA in TaFBA1 OEs plants under ABA and drought conditions (**Figures 11**, **12**). Our previous experiments suggested that upregulation of antioxidant-related genes in OEs plants may be involved in the establishment of strong antioxidant capacity (Zhou et al., 2014). According to the abovementioned results, increasing antioxidant competence and decreasing ROS accumulation may be important mechanisms that underly improved drought tolerance of TaFBA1-overexpressing transgenic plants. This was also confirmed in the transgenic wheat (**Figure S7**).

Given the myriad of evidence from the present and previous studies, we suggest that overexpression of wheat TaFBA1 results in reduced sensitivity to ABA and enhanced stoma opening under drought stress. Increased stoma opening can enhance CO_2_ absorption and lead to maintenance of high-carbon fixation reaction in plant leaves to reduce production of excess reducing equivalents. This further reduces ROS accumulation and leads to improved antioxidant capacity.

## Conclusion

In this study, it was found that wheat TaFBA1 positively regulates plant drought tolerance, which is independent of ABA biosynthesis. It also negatively regulates sensitivity of *Arabidopsis* plants to ABA. TaFBA1-regulated ABA insensitivity may be achieved by regulating ABA-mediated gene expression through interactions with RCAR1 and ABI5. Increased antioxidant competence and decreased ROS accumulation may be important mechanisms that underly improved drought tolerance of TaFBA1-overexpressing transgenic plants.

## Author Contributions

JA designed the experiments. JA, QL, and JY performed the experiments. GZ, ZZ, YWu, and YWang provided assistance on the experiments. WW provided vital advice on the article. JA wrote the manuscript.

## Funding

This research was supported by the National Natural Science Foundation of China (31370304) and by Funds of Shandong “Double Tops” Program.

## Conflict of Interest

The authors declare that the research was conducted in the absence of any commercial or financial relationships that could be construed as a potential conflict of interest.

## Supplementary Material

Figure S1 Confirmation of *TaFBA1* transgenic lines by RT-PCR. **(A)** RT-PCR of TaFBA1 overexpression in *Arabidopsis thaliana* lines and **(B)** *TaFBA1* homozygous gene mutant recovery lines.

Figure S2 ABA levels in WT, TaFBA1-overexpression (OE4, OE5, OE6) and mutant (*atfbw2-4*, *atfbw2-5*, *atfbw2-6*) *Arabidopsis* lines under drought stress.

Figure S3 Phenotypes of WT and recovery (R-4, R-5, R-6) grown and WT, mutant, *TaFBA1*-overexpression *Arabidopsis* plants rehydration ability under drought stress condition. **(A)** Phenotypes of WT and recovery (R-4, R-5, R-6) *Arabidopsis* plants grown under drought stress condition. **(B)** WT, *TaFBA1*-overexpression and **(C)** WT, mutant *Arabidopsis* plants rehydration ability under drought stress condition.

Figure S4 Phenotypes of WT and recovery (R-4, R-5, R-6) *Arabidopsis* plants after ABA treatment.

Figure S5 Interactions of TaFBA1 with RCAR1 were analyzed *via* the LCI assay.

Figure S6 Interactions of AtFBW2 with RCAR1 and ABI5 were analyzed *via* the yeast two-hybrid assay.

Figure S7 The oxidation resistance of CB037, TaFBA1-overexpression (OE2, OE6) and TaFBA1-RNAi (R4, R5) in wheat. **(A)** Phenotypes of CB037, *TaFBA1* overexpression (OE2, OE6) and *TaFBA1*-RNAi (R4, R5) after 10 μM methyl viologen (MV) treatment. **(B)** The chlorophyll and **(C)** MDA contents of CB037, *TaFBA1*-overexpression (OE2, OE6) and *TaFBA1*-RNAi (R4, R5) after 10-μmol methyl viologen (MV) treatment.
