# Supplementary figures and images for: Wheat F-box Protein TaFBA1 Positively Regulates Plant Drought Tolerance but Negatively Regulates Stomatal Closure
Source: Front Plant Sci. 2019 Oct 10;10:1242. doi: 10.3389/fpls.2019.01242 (PMC6795708; doi:10.3389/fpls.2019.01242)

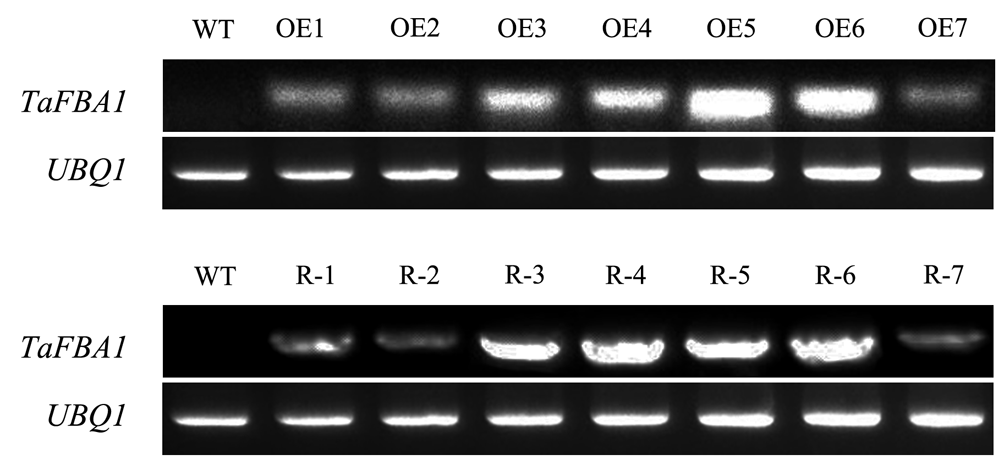

Supplement: Figure S1 — Confirmation of TaFBA1 transgenic lines by RT-PCR. (A) RT-PCR of TaFBA1 overexpression in Arabidopsis thaliana lines and (B) TaFBA1 homozygous gene mutant recovery lines. [file Image_1.tif]

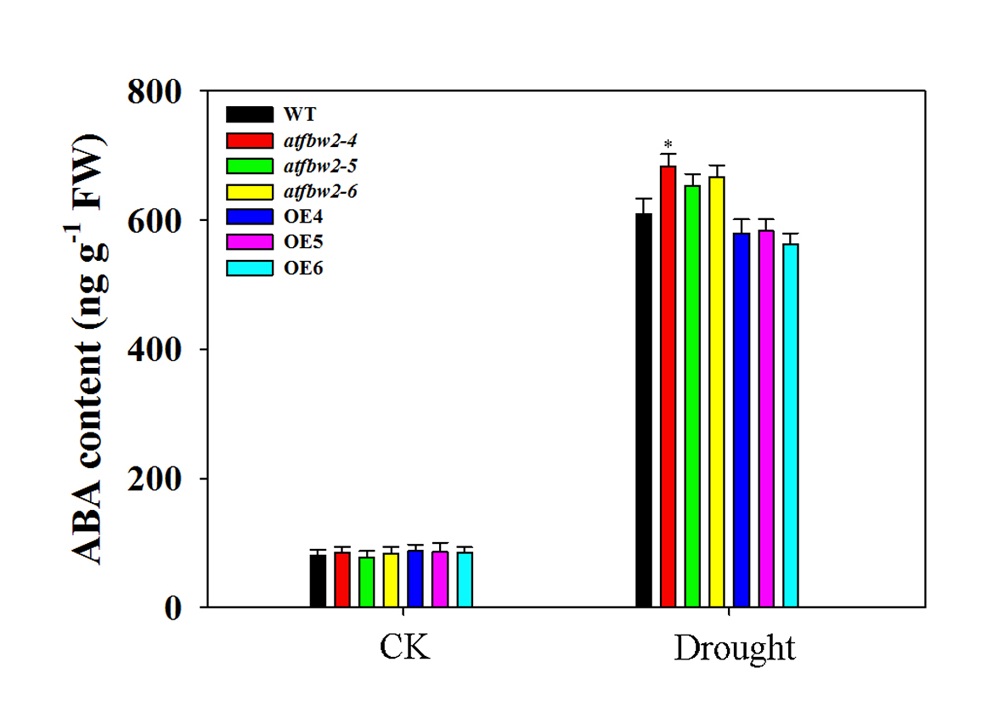

Supplement: Figure S2 — ABA levels in WT, TaFBA1-overexpression (OE4, OE5, OE6) and mutant (atfbw2-4, atfbw2-5, atfbw2-6) Arabidopsis lines under drought stress. [file Image_2.tif]

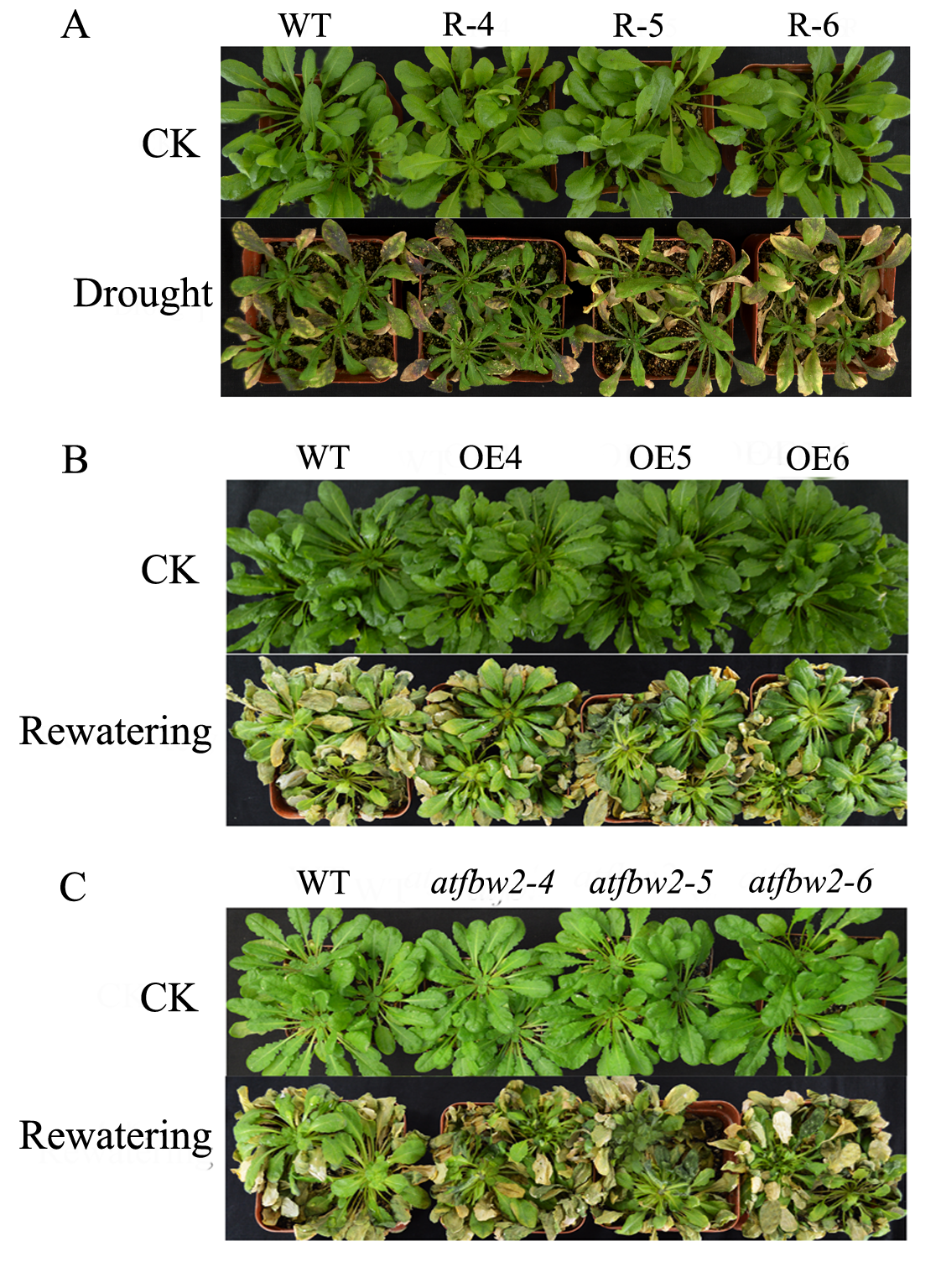

Supplement: Figure S3 — Phenotypes of WT and recovery (R-4, R-5, R-6) grown and WT, mutant, TaFBA1-overexpression Arabidopsis plants rehydration ability under drought stress condition. (A) Phenotypes of WT and recovery (R-4, R-5, R-6) Arabidopsis plants grown under drought stress condition. (B) WT, TaFBA1-overexpression and (C) WT, mutant Arabidopsis plants rehydration ability under drought stress condition. [file Image_3.tif]

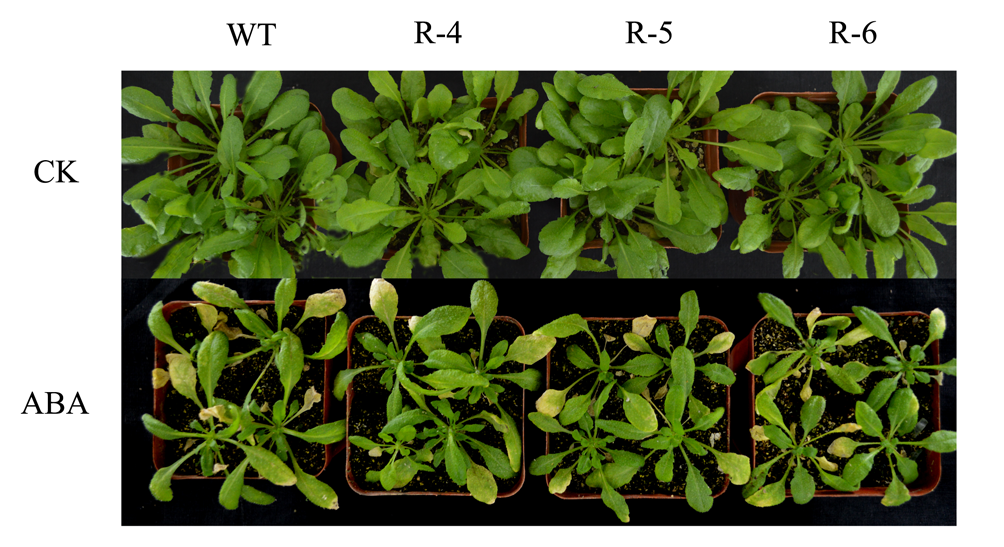

Supplement: Figure S4 — Phenotypes of WT and recovery (R-4, R-5, R-6) Arabidopsis plants after ABA treatment. [file Image_4.tif]

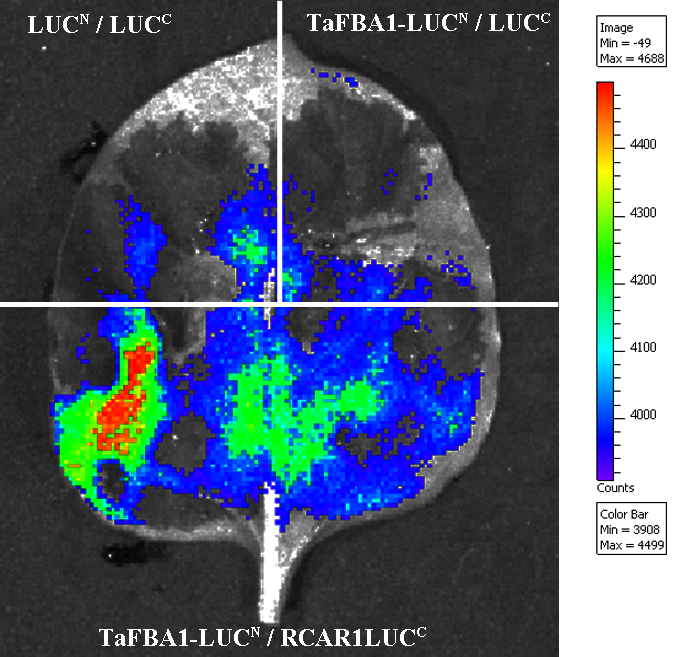

Supplement: Figure S5 — Interactions of TaFBA1 with RCAR1 were analyzed via the LCI assay. [file Image_5.tif]

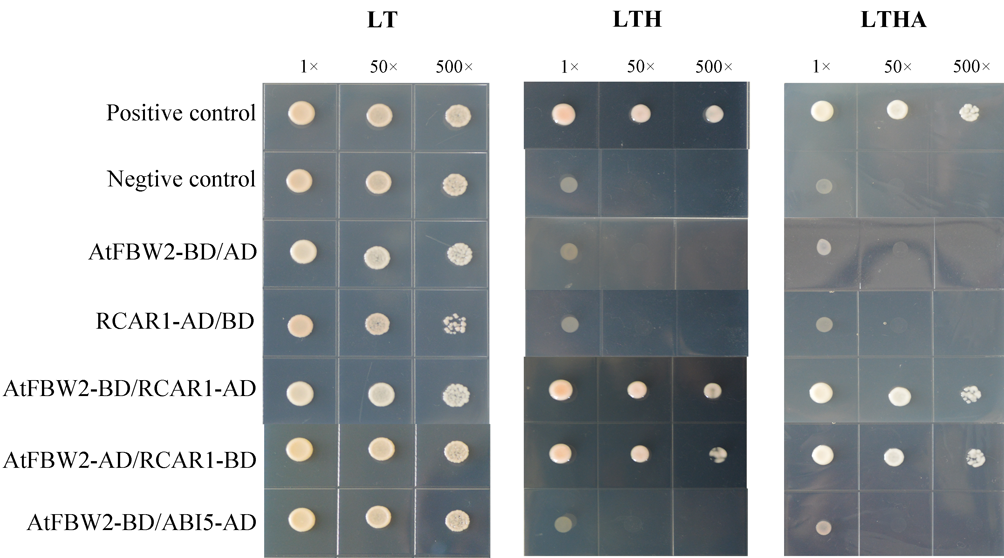

Supplement: Figure S6 — Interactions of AtFBW2 with RCAR1 and ABI5 were analyzed via the yeast two-hybrid assay. [file Image_6.tif]

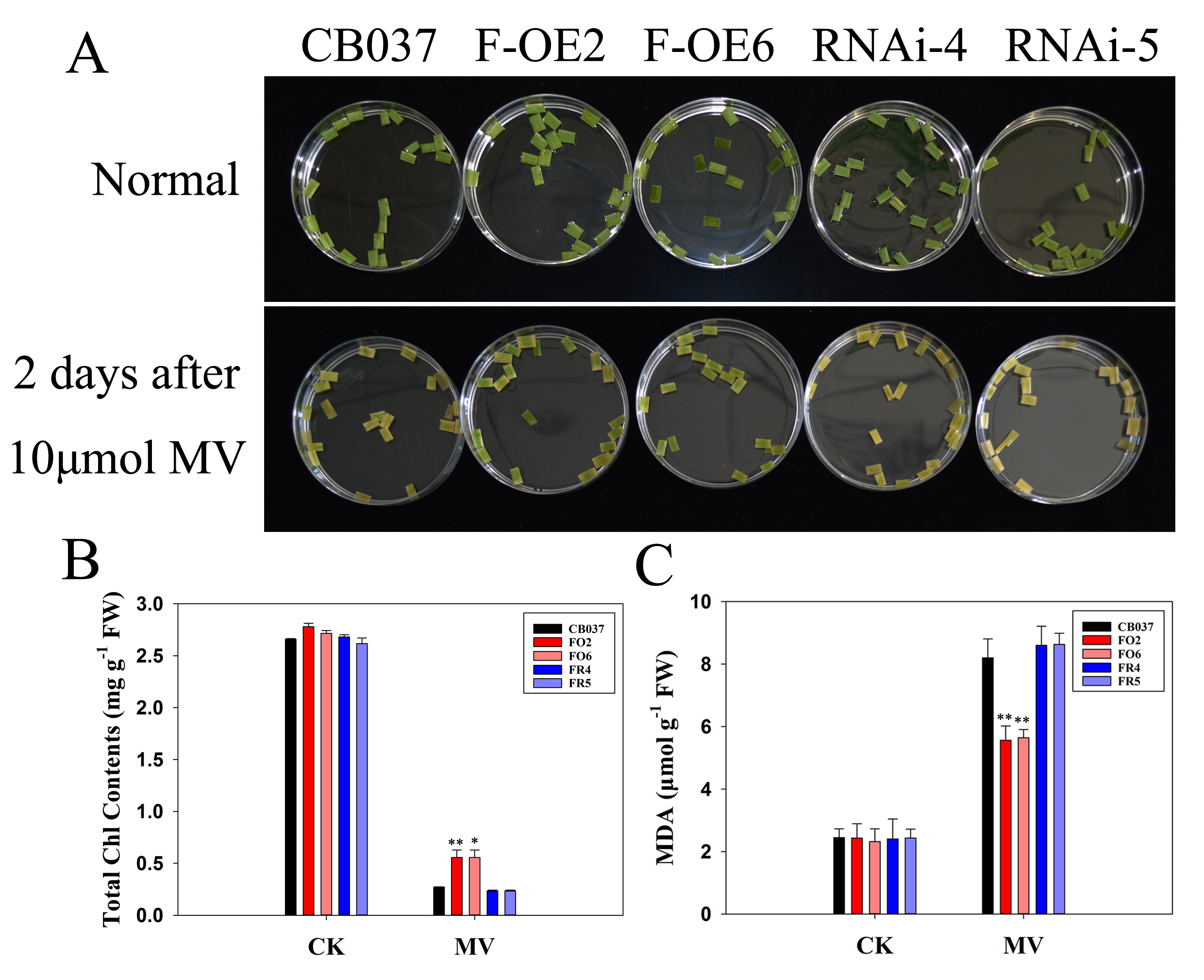

Supplement: Figure S7 — The oxidation resistance of CB037, TaFBA1-overexpression (OE2, OE6) and TaFBA1-RNAi (R4, R5) in wheat. (A) Phenotypes of CB037, TaFBA1 overexpression (OE2, OE6) and TaFBA1-RNAi (R4, R5) after 10 μM methyl viologen (MV) treatment. (B) The chlorophyll and (C) MDA contents of CB037, TaFBA1-overexpression (OE2, OE6) and TaFBA1-RNAi (R4, R5) after 10-μmol methyl viologen (MV) treatment. [file Image_7.tif]
