# Supplementary material for: Wheat F-box Protein TaFBA1 Positively Regulates Plant Drought Tolerance but Negatively Regulates Stomatal Closure
Source: Front Plant Sci. 2019 Oct 10;10:1242. doi: 10.3389/fpls.2019.01242 (PMC6795708; doi:10.3389/fpls.2019.01242)
Supplement: Supplementary file 8 [file Table_1.doc]

**Table S1. Primers used in this study**

| Name | Primer sequence (5’-3’) |
| --- | --- |
| ***qRT-PCR*** | |
| TaFBA1-RT-F | AGCAGCAGAACAAGCCTGACCA |
| TaFBA1-RT-R | ACGTGACGTTGGACAGCCTTTG |
| AtRD29-RT-F | GATGACGAGCTAGAACCT |
| AtRD29-RT-R | CTTGTCCGATGTAAACG |
| AtRAB18-RT-F | GGCGTCTTACCAGAACCGTCCA |
| AtRAB18-RT-R | CCGTATCCTCCTCCTCCCATCG |
| AtABF3-RT-F | AACCGTTCTCAACCTGCAAC |
| AtABF3-RT-R | TTGGAGTCAGATCAGGTGACAT |
| AtABI1-RT-F | TCTCAGGTAGCGAACTATTGTAG |
| AtABI1-RT-R | TGGTCAACGGATAATGGAAGTG |
| AtABI2-RT-F | GATCACAAACCGGATAGGGA |
| AtABI2-RT-R | CCATCGCGTTCTTCTTATGC |
| AtABI3-RT-F | CAGCTTCTGCTATGCCACGTC |
| AtABI3-RT-R | CACAAAATCGCCGGTGTTC |
| AtABI4-RT-F | CTTCCTTAATGGTGGGACCTC |
| AtABI4-RT-R | ACCAGCTAGAGAGTTCAAATCC |
| AtABI5-RT-F | ACAGCAAATGGGAATGGTTGG |
| AtABI5-RT-R | AACTCCGCCAATGCATGTTT |
| AtABA1-RT-F | TCGATGCTTGACTGGGTCCT |
| AtABA1-RT-R | TCGAAACCTGAGACGAAGG |
| AtABA2-RT-F | ATGGCGATGTTAGAGTGGAAG |
| AtABA2-RT-R | AGAATGTGGACCAACGCCTC |
| AtNCED3-RT-F | AGTGTCCTGTCTGAAATCCG |
| AtNCED3-RT-R | ATGTATCCTTCGTCTTCCTCTCC |
| AtGORK-RT-F | CTGGTCATGTTAGATTGCAGTACGA |
| AtGORK-RT-R | CTGTGGAGCAGCCTTTGAAGA |
| AtSLAC1-RT-F | TCCCTAGTTGCTCGGATCAATTTC |
| AtSLAC1-RT-R | GTTGCTACGGATGCTGTTGTCATA |
| AtOST1-RT-F | ACTCCTGCTTACATCGCTCCTGA |
| AtOST1-RT-R | GCTCCAACCAGCATGACATACA |
| AtABCG40-RT-F | TAACCACCACATCGCCTCCG |
| AtABCG40-RT-R | TCGTGGTCGTGTTTTCCCTCGT |
| UBQ1- RT -F | CTTGTGTTGAGGCTTAGAGGAG |
| UBQ1- RT -R | CTTGGGTGAAGACGAGCATAG |
| ***gene amplification*** | |
| TaFBA1-F | CACCGGAGCAGAGATGGAAGAGCA |
| TaFBA1-R | AGTCGCTGATCTCGCTCCTC |
| RCAR1-F | CACCATGATGGACGGCGT |
| RCAR1-R | CTGAGTAATGTCCTGAGAAGCC |
| ABI5-F | CACCATGGTAACTAGAGAAACG |
| ABI5-R | GAGTGGACAACTCGGGTTC |
| ABI2-F | CACCATGGACGAAGTTTCTCC |
| ABI2-R | TTCAAGGATTTGCTCTTGAA |
| ABF3-F | CACCATGGGGTCTAGATTAA |
| ABF3-R | CCAGGGACCCGTCAATGT |
